# Molecular Insight into the Structural Properties of Deep Eutectic Solvents Based on Alkanolamines—A Theoretical and Experimental Study

**DOI:** 10.3390/molecules31081364

**Published:** 2026-04-21

**Authors:** Maciej Śmiechowski, Bartosz Nowosielski, Ingmar Persson, Iwona Cichowska-Kopczyńska, Dorota Warmińska

**Affiliations:** 1 Department of Physical Chemistry, Faculty of Chemistry, Gdańsk University of Technology, ul. Narutowicza 11/12, 80-233 Gdansk, Poland; 2 Maritime Advanced Research Centre, Szczecińska 65, 80-392 Gdansk, Poland; 3 Department of Molecular Sciences, Swedish University of Agricultural Sciences, P.O. Box 7015, SE-750 07 Uppsala, Sweden; 4 Department of Process Engineering and Chemical Technology, Faculty of Chemistry, Gdańsk University of Technology, ul. Narutowicza 11/12, 80-233 Gdansk, Poland

**Keywords:** alkanolamines, deep eutectic solvents, tetraalkylammonium salts, structure, molecular dynamics, ab initio molecular dynamics, large-angle X-ray scattering

## Abstract

Molecular dynamics simulations were performed on 27 deep eutectic solvents (DESs) composed of various hydrogen bond acceptors (HBAs)—tetrabutylammonium bromide (TBAB), tetrabutylammonium chloride (TBAC), and tetraethylammonium chloride (TEAC)—combined with different hydrogen bond donors (HBDs)—3-aminopropan-1-ol (AP), 2-(methyl-amino)ethanol (MAE), and 2-(*n*-butylamino)ethanol (BAE). Radial distribution functions (RDFs) were computed from the simulation trajectories to probe the microscopic structure of these DESs. The effects of HBA/HBD molar ratio, alkyl chain length, anion type, and the amine group’s substitution on the structural organization of the DESs were systematically investigated. Moreover, the influence of water addition on the structural properties of selected DESs (TBAB with AP, MAE, or BAE at a 1:6 molar ratio) was explored. These structural features were then correlated with previously reported experimental data. To complement the classical simulations, ab initio molecular dynamics simulations were conducted on the same TBAB-based systems, enabling the analysis of electronic structure phenomena, including RDFs, dipole moment distributions, and charge transfer. Furthermore, experimental large-angle X-ray scattering (LAXS) data collection and analysis were performed in terms of the simulated structural data. This multi-scale approach provides a detailed understanding of the structural and electronic characteristics governing the behavior of alkanolamine-based DES.

## 1. Introduction

In recent years, deep eutectic solvents (DESs) have been gaining recognition as a potential environmentally friendly alternative to traditional organic solvents [1]. These solvents consist of two or more components, most often hydrogen bond acceptors (HBAs) and hydrogen bond donors (HBDs) that interact through hydrogen bonds (H-bonds) and/or electrostatic interactions, resulting in a liquid with a lower melting point than the individual components [2]. According to Martins et al., the term “deep eutectic solvent” can only be used to refer to an eutectic mixture that has a negative melting point deviation from the ideal eutectic and maintains a liquid state at a specific operating temperature for a given composition [3].

Due to their overlapping general characteristics, DESs are often treated as a subset of ionic liquids (ILs). These common features typically include high thermal stability, low volatility, minimal vapor pressure, and adjustable polarization. However, in comparison to ILs, DESs are cheaper, easier to prepare, less toxic, and often biodegradable [4]. Moreover, as with ILs, the properties of DESs can be tuned by changing the composition or components of these liquids. This makes them ideal solvents for several applications such as metallurgy [5], electrodeposition [6], extraction [7], gas separation [8,9], and biomass processing [10].

Currently, in parallel with experimental research on DESs in terms of their physicochemical properties and suitability for a specific process, theoretical studies are being carried out, mainly through molecular dynamics (MD) simulations and quantum mechanical methods. Most commonly, these computational techniques are used to obtain direct information about the structure of DESs, which is difficult or impossible to obtain using experimental techniques (such as Fourier transform infrared spectroscopy (FTIR) or nuclear magnetic resonance spectroscopy (NMR)) and can help elucidate the properties of DESs [4,11,12,13,14].

The first works employing MD simulation were invaluable in explaining the mechanism of DES formation and its freezing point. It has been shown that in the case of choline chloride-based DESs, strong H-bonds formed between the chloride anion and the hydroxyl group of HBD are responsible for the melting point depression of the solvent [15,16]. Similar results were obtained for DESs consisting of methyltriphenylphosphonium bromide (MTPB) or tetra-*n*-butylammonium bromide (TBAB) as the HBA and ethylene glycol (EG) or glycerol (Gly) as the HBD [17]. Furthermore, Migliorati et al. stated that groups other than the halogen anion in HBA have a significant impact on the hydrogen bond network in DESs [18]. They compared the structures of DESs based on choline chloride (ChCl) and benzyltrimethyl-ammonium chloride (BTMA). They found that the –OH group in ChCl forms a hydrogen bond with HBD molecules, leading to a three-dimensional arrangement of all components of the mixture. This is very different from the hydrogen bond network structure that is formed in DESs based on benzyltrimethylammonium chloride, where the cation is unable to form such hydrogen bonds due to the lack of a hydroxyl group. The work of Ferreira et al. showed that the HBD also has a significant impact on the network of hydrogen bonds formed in DESs [19]. According to their result, a higher number of hydroxyl groups in the HBD molecule of a DES leads to a higher level of hydrogen bonding of both HBA–HBD and HBD–HBD types. Moreover, the closer the functional groups in the HBD are to each other, the fewer but stronger hydrogen bonds are formed in the DES. Simulations performed by Khorasani et al. showed that DES formation is driven by strong hydrogen bonds between polyethylene glycol’s terminal hydroxyl groups and TBAB’s bromide anions [20]. Rozas et al. demonstrated that depending on the HBD, its functional groups can also interact with the HBA differently [21]. In the 1,8-cineole (CN)/malic acid (MA) mixture, there was a minimal difference between the hydrogen bonds formed by the central and terminal hydroxyl groups of HBD. In contrast, in the lactic acid-based DES, the interactions through the –COOH group were distinctly stronger than those through the –OH group. The study involving a DES containing methyltriphenylphosphonium bromide and monoethanolamine (MEA) revealed an almost fivefold stronger interaction between Br^−^ and the hydroxyl group compared to the amine group [22]. Another factor, differing from the type of HBA and HBD and also influencing the structure of deep eutectic solvent, i.e., the HBA/HBD molar ratio, was studied by Pour et al. and He et al. [23,24]. Hydrogen bond analysis by Pour et al. indicated that an increase in glucose content in choline chloride-based DESs led to a decrease in the interactions between the choline cation and the chloride ion, while increasing the interactions between sugar and Cl^−^. This phenomenon was correlated by the authors with the higher experimental viscosity of DES with increased glucose content [23]. He et al. studied DES consisting of betaine and 1,2-propanediol and observed a weakening of the interactions between HBA with HBD with increasing glycol content [24]. Therefore, the opposite conclusions obtained for different DESs suggest that the influence of the HBA/HBD molar ratio may be different and depends on the components constituting the DES. Recently published studies by Lane et al. have shown that the molecular size of both the HBA and HBD significantly affects the nature of hydrogen bonding in DESs. In particular, mixtures of L-leucic acid with larger alkylammonium cations and smaller anions exhibit stable hydrogen bonding interactions [25].

In some processes, water is intentionally added to a deep eutectic solvent to modulate its physicochemical properties, especially mass transfer properties, such as viscosity. This affects the structure of the solvent, and several MD studies of aqueous DES solutions have been carried out. Shah et al. investigated interactions within aqueous ChCl/urea (1:2) solutions [26]. The study revealed a preference for Cl^−^ to be more readily hydrated by water than coordinated by urea or the choline cation. Moreover, the influence of water content was categorized into three ranges based on the mass fraction of H_2_O. In the initial range (w_H2O_ < 5%), the number of hydrogen bonds increased, reaching a maximum at w_H2O_ ≈ 2.5%. Moving into the subsequent range (from 5% to 25% water mass fraction), choline chloride and urea molecules exhibited increased hydration, and both the anion and urea demonstrated low diffusivity. In the final range (with a water mass fraction exceeding 25%), the anion and urea displayed high diffusivity. Zhekenov et al. investigated the change in the number of hydrogen bonds upon the addition of water to three DESs, namely reline, ethaline, and glyceline [27]. Regardless of DES, an increase in H_2_O content resulted in an increase in hydrogen bonds between water molecules and both the HBA and HBD. However, the other pairs, such as HBD–choline ion, HBD–chloride ion, and HBD–HBD, exhibited a decrease in the number of hydrogen bonds with the addition of water. This suggested a higher affinity of DES components towards water molecules than other species present in the system. Araneva et al. and Monteiro et al. studied the effect of water on DESs composed of ChCl or betaine with EG, 1,2-propanediol, 1,3-propanediol, and 1,4-butanediol [28,29]. Similar to previous findings, an elevation in water content led to a reduction in the number of hydrogen bonds between the HBA and HBD. The systems containing ethylene glycol exhibited the fewest hydrogen bonds between the HBD and water molecules. This was attributed to the proximity of the two hydroxyl groups in ethylene glycol, facilitating the simultaneous formation of hydrogen bonds with a single water molecule. This effect also contributed to a lower number of hydrogen bonds between HBD and water in 1,2-propanediol compared to 1,3-propanediol. Recently, Monteiro et al. studied the transition from a DES−water system to an aqueous solution in betaine/glycerol DES with increasing addition of water [30]. It was found that the studied DES shows a continuous structural transition with increasing water content, exhibiting a major structural transformation at 70 mol% water. Notably, this structural transformation manifested in the second coordination shell of the DES components. Similar observations were reported by Garmroodi et al., who studied aqueous solutions of DES composed of menthol and thymol, further supporting the notion that in a water-rich region, DES constituents are separately hydrated [31].

Ab initio molecular dynamics (AIMD) simulations combine the quantum mechanical description of the electronic structure with the classical evolution of the nuclei on the electronic potential surface [32]. To date, AIMD simulations have been used to study a limited selection of DES systems, particularly those based on choline chloride [33,34,35,36,37,38,39,40,41]. Some studies confirm the micro-heterogenous structure of DES systems [36,40]. Examining DES + water systems, it was found again that reline and ethaline respond differently to the addition of H_2_O. In reline, existing urea–Cl^−^ H-bonds are partially substituted by H_2_O–Cl^−^ ones, leading to the significantly increased mobility of both urea and chloride. In contrast, in ethaline, the H-bonds formed by HBD are only very slightly perturbed by the introduction of water [36,37].

The structure of the system envisaged by MD simulations can be obtained experimentally by scattering techniques, including large-angle X-ray scattering (LAXS). In the context of DES systems, LAXS was most often used together with small-angle X-ray scattering (SAXS) to determine the medium-range order in the liquid samples at low values of the scattering vector, *q* [42]. Only in a handful of studies was the spectrum measured at high enough scattering angles—and thus high *q* values—that gave access to short-range intermolecular interactions in DES [43,44]. By investigating tetrabutylammonium bromide–imidazole DES, it was found that the intermolecular contacts at 3–5 Å dominate the scattering intensity, but no decomposition into individual pairs was attempted [43]. In a recent study of choline chloride mixtures with simple carboxylic acids, the scattering curves were modeled using empirical potential structure refinement, thus allowing access to individual radial distribution functions in the system. It was shown that both choline cation and the acid molecule compete in donating H-bonds to Cl^−^ anions, with two H-bonds formed on average to each chloride [44].

In this work, we performed molecular dynamics simulations on 27 DESs. These solvents were composed of tetrabutylammonium bromide (TBAB), tetrabutylammonium chloride (TBAC), and tetraethylammonium chloride (TEAC) as the HBA, and 3-aminopropan-1-ol (AP), 2-(methylamino)ethanol (MAE), and 2-(*n*-butylamino)ethanol (BAE) as the HBD. Radial distribution functions (RDFs) were obtained from the simulation trajectories to probe the microscopic structure of these DESs. Our investigation systematically explored the effects of HBA/HBD molar ratio, alkyl chain length in both HBA and HBD, anion type, and the order of amine group on the structural organization of the DES. Furthermore, we also delved into the influence of water addition on the structural properties of selected DESs (TBAB with AP, MAE, or BAE at a molar ratio of 1:6). These structural features were then correlated with previously published experimental data. To complement the classical simulations, we conducted ab initio molecular dynamics simulations of the solvents composed of TBAB and the three HBD molecules (AP, MAE, or BAE) at a molar ratio of 1:6, to gain insight into the phenomena related to the electronic structure of such systems. We analyzed dipole moment distributions, infrared spectra, and charge transfer phenomena in the system. For the same systems, we also performed LAXS data collection and analysis in the angle scattering range of 1–130 degrees, giving experimental RDFs. This multi-scale approach provides a detailed understanding of the structural and electronic characteristics governing the behavior of alkanolamine-based DESs, with practical implications for the design and optimization of these solvents in various applications. In particular, such systems are of considerable interest in CO_2_ capture, as both the molecular structure of the solvent components and the resulting intermolecular interactions may significantly affect absorption performance. Alkanolamine-based DESs are especially attractive in this context because they combine the presence of amino groups, which can promote interactions with CO_2_, with hydroxyl functionalities that contribute to the formation and modulation of the hydrogen bonding network. To our knowledge, molecular dynamics studies of this class of DESs remain very limited and have so far focused primarily on systems based on choline chloride and conventional alkanolamines [22,45]. Therefore, the novelty of this work lies in extending these studies to DESs consisting of tetraalkylammonium halides and less-studied alkanolamines, namely 2-(methylamino)ethanol (MAE), 2-(*n*-butylamino)ethanol (BAE), and 3-aminopropan-1-ol (AP), thereby providing new insights into structure–property relationships relevant to carbon dioxide capture applications.

## 2. Results and Discussion

For molecular dynamics simulations, an initial validation of the suitability of the applied methodology in reproducing DES properties was provided by computing average liquid densities and comparing them to the experimental results [46,47,48]. The obtained densities were found to be quite accurate, with mean relative error of ca. 1.5%. The calculated densities were systematically lower than the experimental ones; however, the experimentally observed trends with changing salt content and/or alcoholamine type were properly reproduced. A detailed comparison is provided in the Supplementary Material (Table S1).

**Figure 1:**
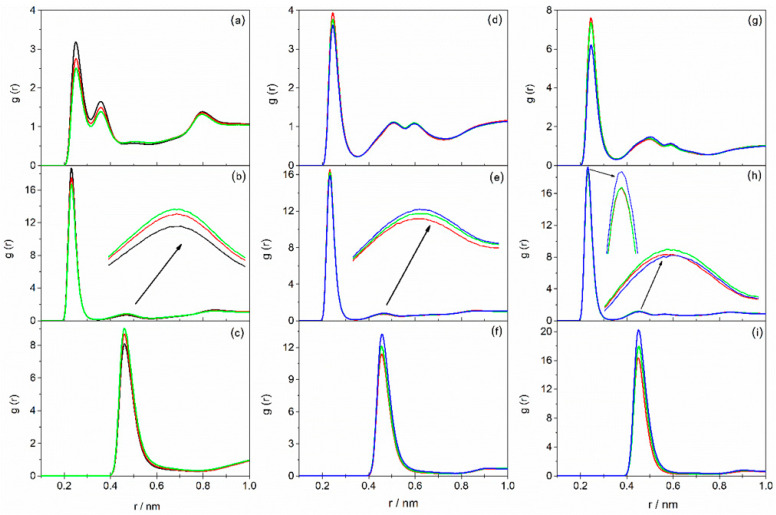
Radial distribution functions as a function of interatomic distances: Br^−^⋯H–N (**a**,**d**,**g**); Br^−^⋯H–O (**b**,**e**,**h**); N^+^⋯Br^−^ (**c**,**f**,**i**) for DESs based on TBAB containing AP (**a**–**c**), MAE (**d**–**f**), BAE (**g**–**i**) with various HBA–HBD molar ratios: black—1:4; red—1:6; green—1:8; blue—1:10.

**Figure 2:**
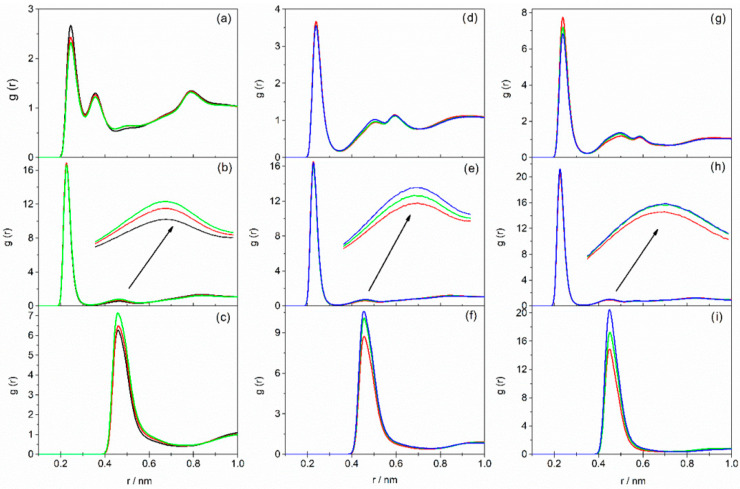
Radial distribution functions as a function of interatomic distances: Cl^−^⋯H–N (**a**,**d**,**g**); Cl^−^⋯H–O (**b**,**e**,**h**); N^+^⋯Cl^−^ (**c**,**f**,**i**) for DESs based on TEAC containing AP (**a**–**c**), MAE (**d**–**f**), BAE (**g**–**i**) with various HBA–HBD molar ratios: black—1:4; red—1:6; green—1:8; blue—1:10.

**Figure 3:**
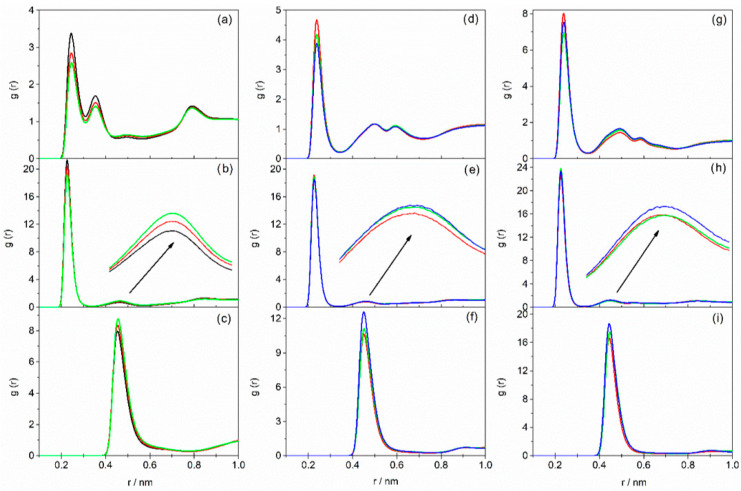
Radial distribution functions as a function of interatomic distances: Cl^−^⋯H–N (**a**,**d**,**g**); Cl^−^⋯H–O (**b**,**e**,**h**); N^+^⋯Cl**^−^** (**c**,**f**,**i**) for DESs based on TBAC containing AP (**a**–**c**), MAE (**d**–**f**), BAE (**g**–**i**) with various HBA–HBD molar ratios: black—1:4; red—1:6; green—1:8; blue—1:10.

Anion solvation significantly influences the microstructure of DESs based on tetraalkylammonium halides, which is also crucial for the formation of DESs [25]. In Figure 1, Figure 2 and Figure 3, we present structural data on the studied pure DES systems obtained from MD in the form of radial distribution functions (RDFs). This allows for a simultaneous comparison of how the HBA cation and anion, the HBD, and the HBA–HBD molar ratio impact the structure of the DES. For each series of DESs containing the same alkanolamine, the lowest ratio of HBA to HBD was chosen as the minimum alkanolamine content at which a stable liquid could be obtained at room temperature.

Figure 1 shows the radial distribution functions for DESs containing TBAB at various salt-to-alkanolamine molar ratios. Specifically, Figure 1a,d,g illustrate the effect of the HBA–HBD molar ratio on the Br^−^⋯H–N interaction, while Figure 1b,e,h depict the corresponding impact on the Br^−^⋯H–O interaction.

In the RDF plots for DESs based on AP, two peaks are observed for the Br^−^⋯H–N interaction within the first coordination sphere of the bromide ion, arising from the presence of the –NH_2_ group in the alkanolamine molecule. In contrast, DESs based on MAE or BAE exhibit only a single peak for this interaction, with a maximum at 0.244 nm. For DESs based on TBAC and TEAC, similar trends to those observed for TBAB-based liquids were seen and are presented in Figure 2 and Figure 3. Detailed numerical data for all systems—specifically, peaks representing anion interactions with cation, as well as with the amino or hydroxyl groups of the alkanolamine, along with the coordination number of the halide ion (considering the first coordination sphere)—are compiled in Tables S2–S4 in the Supplementary Material.

As can be seen, the intensity of the peaks corresponding to the X^−^⋯H–N interaction in the first coordination sphere of the halide ion decreased with increasing HBD content in the DES for all the studied systems (Figure 1a,d,g, Figure 2a,d,g and Figure 3a,d,g), while the coordination number of X^−^ increased (Table S2). Therefore, with a higher alkanolamine content, a larger number of hydrogen bonds was formed, but they were weaker.

The analysis of RDF plots representing X^−^⋯H–O interactions leads to similar conclusions (Figure 1b,e,h, Figure 2b,e,h and Figure 3b,e,h). The exception is observed for DESs containing BAE, where the intensity of the peak corresponding to X^−^⋯H–O interactions in the first coordination sphere of the halide ion is the highest at the HBA–HBD molar ratio of 1:10. Nevertheless, it seems reasonable to conclude that with increasing HBD content, the hydrogen bond between HBA and HBD becomes less favored. This is further confirmed by the analysis of the peak corresponding to X^−^⋯H–O interactions in the second coordination shell of the halide ion (second peak at around 0.46 nm in Figure 1b,e,h, Figure 2b,e,h and Figure 3b,e,h). The increasing intensity of this second peak with rising AP or MAE content in the DES, along with a simultaneous decrease in the intensity of the first-shell peak, may indicate a migration of alkanolamine molecules from the first to the second coordination shell, resulting in weakening of the hydrogen bonding between the salt and alkanolamine. This effect is less pronounced in DESs based on BAE.

The weakening of hydrogen bonding between DES components with increasing HBD content has also been previously observed in TBAB/EG systems. In those systems, the addition of ethylene glycol resulted in a diminished height of the Br^−^⋯EG peak in RDF plots [49]. This reduction has been attributed to stronger self-association among EG molecules, which subsequently competes with bromide–ethylene glycol interactions. For the studied DESs, the formation of stronger hydrogen bonds between alkanolamine molecules and weaker interactions between HBA and HBD upon increasing alkanolamine content is also indicated by the increased intensity of the N^+^⋯X^−^ peak in RDFs (Figure 1c,f,i, Figure 2c,f,i and Figure 3c,f,i). The decrease in the halide ion’s coordination number (obtained by integrating the N^+^⋯X^−^ peak area) as alkanolamine content increases is attributed to the dilution of the salt and a reduced tendency to form ion pairs at lower salt concentrations.

**Figure 4:**
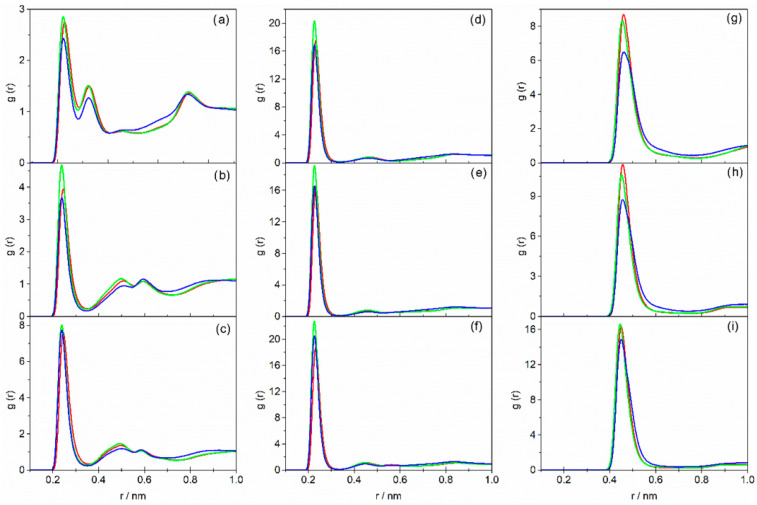
Radial distribution functions as a function of interatomic distances: X^−^⋯H–N (**a**–**c**); X^−^⋯H–O (**d**–**f**); N^+^⋯X^−^ (**g**–**i**) for DESs based on AP (**a**,**d**,**g**), MAE (**b**,**e**,**h**), BAE (**c**,**f**,**i**) containing red—TBAB; green—TBAC; and blue—TEAC with an HBA–HBD molar ratio of 1:6.

**Figure 5:**
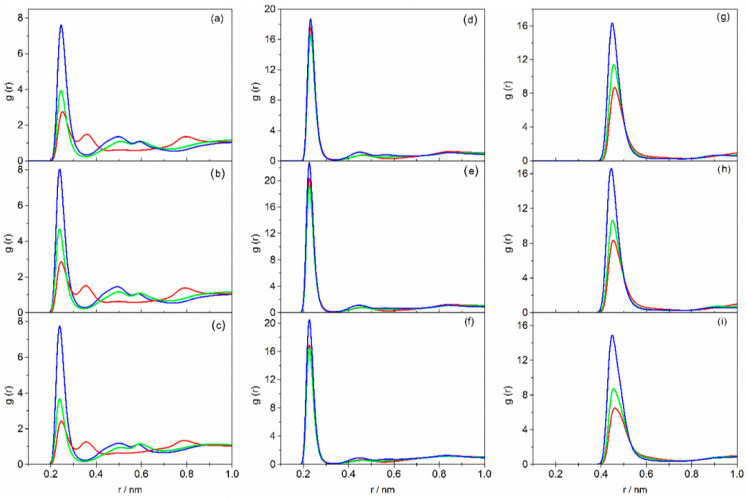
Radial distribution functions as a function of interatomic distances: X^−^⋯H–N (**a**–**c**); X^−^⋯H–O (**d**–**f**); N^+^⋯X^−^ (**g**–**i**) for DESs based on TBAB (**a**,**d**,**g**), TBAC (**b**,**e**,**h**), and TEAC (**c**,**f**,**i**) containing red—AP; green—MAE; blue—BAE with an HBA–HBD molar ratio of 1:6.

To better compare the effect of HBA and HBD on the DES structure, Figure 4 and Figure 5 show two different views of RDFs shown in Figure 1, Figure 2 and Figure 3 with a chosen fixed HBA–HBD molar ratio of 1:6. Figure 4 illustrates the influence of the hydrogen bond acceptor on the structure of studied DESs, while Figure 5 presents the hydrogen bond acceptor effect.

When comparing the RDFs of mixtures containing TBAC (green lines) and TBAB (red lines), higher intensity peaks are observed in the case of TBAC-based DESs in interactions involving both the –NH group (Figure 5a–c) and the –OH group (Figure 5d–f) of the HBD. This trend is independent of the alkanolamine used, and the peak maxima are systematically shifted towards shorter distances in the TBAC systems. These features indicate stronger hydrogen bond interactions between the alkanolamine and TBAC compared to TBAB. Nevertheless, the overall number of hydrogen bonds is lower in TBAC-based DESs, as reflected by the coordination numbers given in Tables S2–S4. Similar behavior was reported by Migliorati and D’Angelo for DESs composed of urea and choline chloride or fluoride [50]. In that study, the stronger F^−^⋯H–N interaction peaks were attributed to the lower molar mass of the fluoride anion relative to the chloride anion.

When analyzing the present results, it is worth noting that the observed differences in interaction strengths between the studied alkanolamines and TBAB or TBAC are relatively small, especially in the case of AP- and BAE-based DESs. This is consistent with experimental viscosity values in these systems, which are only slightly higher for liquids containing TBAB [46,47,48]. If hydrogen bond strength alone was considered, a higher value could be expected for TBAC-based DESs. Since this is not the case, it can be concluded that in the studied DES systems characterized by slight differences in HBA–HBD interactions, the molar mass and ionic radius of the anion play a decisive role in determining the DES viscosity. The molar mass of the salt anion also affects the density and refractive index of the liquid. This conclusion is supported by the RDF analysis of cation–anion interactions shown in Figure 4g–i. In general, for all liquids studied, the peaks corresponding to N^+^⋯X^−^ interactions are broader in the case of DESs containing TBAB, and their maxima are shifted to larger distances compared to TBAC-based systems. This indicates that in the investigated DESs, a larger number of N^+^⋯Br^−^ interactions are formed relative to N^+^⋯Cl^−^ interactions (Tables S2–S4), although the former are weaker. Consequently, in TBAC-based DESs—also considering the slightly stronger interactions between the salt and the alkanolamine discussed above—a higher degree of structural organization is achieved. Nevertheless, due to the higher mass of the Br^−^ ion, DESs containing TBAB exhibit higher values of density and refractive index, as reported previously [46,47,48]. 

Further analysis of Figure 4, which shows the RDFs for DESs based on TBAC (green line) and TEAC (blue line), allows us to evaluate the influence of the alkyl chain length in the HBA molecule on the DES structure. Regardless of the alkanolamine used or the interaction considered, the RDF peak maxima appear at the same distances; however, the signals are lower and more diffuse for TEAC-based DESs. As a result, the coordination numbers of chloride ions in TEAC-based DESs are larger compared to the coordination numbers in systems containing TBAC (Tables S3 and S4). It can therefore be assumed that the smaller size of the TEA^+^ cation compared to the TBA^+^ cation favors the formation of more hydrogen bonds between the chloride anion and the alkanolamine molecules, as well as more electrostatic interactions between the chloride anion and the cation. Consequently, the DESs based on TEAC—an HBA with a shorter alkyl chain—exhibit a more ordered structure, which is reflected in their experimentally determined physical properties [46,47,48]. In particular, both the density and the refractive index of TEAC-based deep eutectic solvents are higher than those of systems containing TBAC. The higher viscosity observed for TBAC-based DES, despite their lower degree of structural organization, is due to the increased flow resistance associated with the movement of the larger TBA^+^ cation compared to the smaller TEA^+^ ion. Similar trends were reported by Salehi et al. in the case of DES containing tetra-*n*-butylammonium, tetra-*n*-heptylammonium, and tetra-*n*-octylammonium chloride [51].

According to our results, the hydrogen bond donor has the most significant influence on the structural properties of DESs and, consequently, on their physicochemical characteristics. This is illustrated in Figure 5, which presents the RDFs obtained for DES containing the studied salts at a molar ratio of HBA to HBD of 1:6. The most pronounced differences are observed in the interactions between the salt anion (X^−^) and the –NH group of the alkanolamine (Figure 5a–c). As noted above, in DES based on AP, two distinct peaks correspond to the X^−^⋯H–N interaction. This is attributed to the formation of hydrogen bonds by each of the two hydrogen atoms in the primary amine AP. Naturally, such a phenomenon is not observed in DESs containing secondary alkanolamines, i.e., MAE and BAE.

As shown in the RDF plots, the peak heights increase in the order AP < MAE < BAE, indicating progressively stronger interactions between the salt and the amine alcohol, with weakest interactions observed for AP-based systems and the strongest for those containing BAE. This trend is further supported by the X^−^⋯H–N distances, which are shorter for DESs based on MAE and BAE and longer for AP-containing systems. Interestingly, despite these weaker individual interactions, AP-based DESs exhibit the highest coordination numbers (Tables S2–S4), suggesting that AP forms a larger number of hydrogen bonds that are collectively more complex, though individually weaker, than those formed with MAE or BAE. This structural picture is consistent with the experimentally determined viscosities [46,47,48]. AP-based DESs exhibited the highest viscosities, while MAE-containing systems showed the lowest viscosities, and coordination numbers were also observed in this study.

Figure 5d–f illustrates the effect of the HBD on the X^−^⋯H–O interaction. In general, the peaks corresponding to this interaction are higher than those for the X^−^⋯H–N interaction, which means that hydrogen bonds formed by the hydroxyl group of the alkanolamine are stronger than those involving the amine group. Furthermore, higher peak heights for the X^−^⋯H–O interactions are observed in the BAE-based DES compared to almost identical peak heights found for systems containing MAE or AP. At the same time, BAE-based DESs exhibit the lowest coordination numbers, suggesting a less compact structure. This behavior can be attributed to steric hindrance resulting from the longer alkyl chains present in BAE. Additional support for this interpretation is provided by the highest peak intensities observed for N^+^⋯X^−^ interactions (Figure 5g–i) in BAE-based DES. These structural features are directly reflected in the experimentally observed densities, which are the lowest for BAE-based among the systems studied [46,47,48].

Since the coordination environment of the halide ion seems to have a decisive effect on the DES structure, we provide a representative snapshot of the immediate surroundings of the bromide ion in the TBAB/MAE 1:6 system in Figure S1 in the Supplementary Material. In this instantaneous frame, the bromide ion is coordinated by the solvent via three Br^−^⋯H–N/O hydrogen bonds, while simultaneously remaining in close contact with two TBA cations with alkyl chains displaced in a way that minimizes the Br^−^⋯N^+^ distance. The coordinated solvent molecules are interconnected with hydrogen bonds as well.

**Figure 6:**
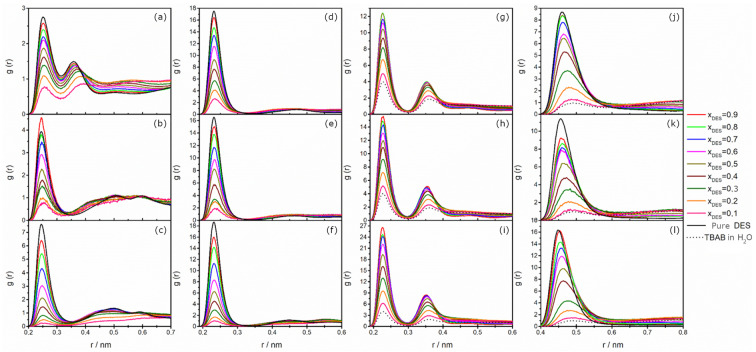
Radial distribution functions as a function of interatomic distances: Br^−^⋯H–N (**a**–**c**); Br^−^⋯H–O (alkanolamine) (**d**–**f**); Br^−^⋯H–O (H_2_O) (**g**–**i**); and Br^−^⋯N^+^ (**j**–**l**) for aqueous solutions of DES based on TBAB with an HBA–HBD molar ratio of 1:6 containing AP (**a**,**d**,**g**,**j**); MAE (**b**,**e**,**h**,**k**); and BAE (**c**,**f**,**i**,**l**).

The effect of water on a TBAB-based DES structure is illustrated in Figure 6. The plots for Br^−^⋯H–N interactions are shown in the first column (Figure 6a–c). For aqueous TBAB/AP 1:6 (Figure 6a), monodentate binding is still more preferable, as in the pure DES, and both peaks decrease in intensity with increasing water addition. Accordingly, coordination numbers decrease (see Table S5 in the Supplementary Material) as alkanolamine is displaced by water from bromide’s solvation shell. It is worth noting that the addition of water also causes opposite trends in the positions of the two peaks: while the first shifts slightly towards shorter distances, the second one shifts more considerably towards longer distances. It shows two concerted phenomena: with the increasing water content, the entire solvation shell of Br^−^ becomes slightly more compact, but also the second amine hydrogen is more readily available to engage in the hydrogen bond network due to smaller size of water molecules acting as hydrogen bond acceptors in this case, which causes the elongation of the second N–H bond.

For MAE- and BAE-based DESs, where only one ammonium proton is available, the intensities of the first peaks steadily decrease along with coordination numbers, with a slight shift to longer distances. This clearly shows that with the addition of water, interactions between DES components become progressively weaker. However, for MAE-based DES at *x*_DES_ = 0.9, the intensity of the peak is higher than for pure DES, which demonstrates that in this particular case, a small addition of water helps to strengthen Br^−^⋯H–N interactions.

Analysis of the Br^−^⋯H–O (alkanolamine) and Br^−^⋯N^+^ RDFs (Figure 6d–f and Figure 6j–l, respectively) also reveals a consistent weakening of the interactions between DES components with increasing water content, regardless of the system studied. This weakening is reflected by the reduction in peak intensity and the shift of the peak position at maximum toward longer distances. Loosening of the DES structure due to the introduction of water molecules was also found by Fetisov et al. for a choline chloride–urea DES [36].

The Br^−^⋯H–O (H_2_O) interactions between bromide and water hydrogen are presented in Figure 6g–i. For all studied DES, two well-defined peaks in the RDFs indicate the formation of two hydration shells. In addition, the peak intensities of the Br^−^⋯H–O (H_2_O) RDFs are higher than in other RDFs, reflecting that hydrogen bonds to bromide in all studied systems and indicating that DES–H_2_O interactions are comparatively the strongest. Panda and Bhargava made similar observations of water mixtures of DESs composed of TBAC coupled with glycerol or ethylene glycol [52]. With the addition of water, peak intensities and coordination numbers decreased, mostly due to the decreasing concentration of Br^−^ anions in the system.

**Figure 7:**
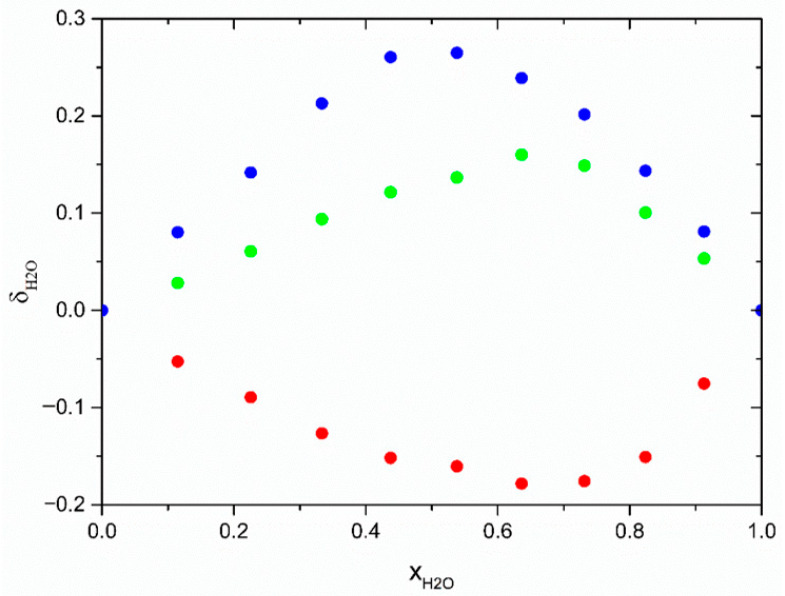
Preferential solvation index of water in the Br^−^ first solvation shell for aqueous solutions of DES based on TBAB with an HBA–HBD molar ratio of 1:6 containing AP (red); MAE (green); and BAE (blue).

Using data from the Br^−^⋯H–N, Br^−^⋯H–O (alkanolamine), and Br^−^⋯H–O (H_2_O) RDFs, we also calculated the preferential solvation index of water in the Br^−^ first solvation:

$$\delta_{H_{2}O}=x_{H_{2}O}\left({\mathrm{Br}}^{-}\right)-x_{H_{2}O}\left(\mathrm{bulk}\right)$$

where $x_{H_{2}O}\left({\mathrm{Br}}^{-}\right)$ and $x_{H_{2}O}\left(\mathrm{bulk}\right)$ are mole fractions of water molecules in the first solvation shell and in the entire solution, respectively. This index reveals interesting differences between the studied aqueous DES systems (see Figure 7). While for AP, the preferential solvation index is negative with a minimum at $x_{H_{2}O}\approx 0.64$, for both MAE and BAE, it is uniformly positive, with a maximum at $x_{H_{2}O}\approx 0.64$ and $x_{H_{2}O}\approx 0.54$, respectively. This means that while in AP, the first solvation shell of bromide contains less water than expected from random coordination, in MAE and BAE, the situation is reversed, and the water content is higher than expected. This is most probably related to the fact that bulkier secondary amines are displaced faster than the linear-chain primary amine from the solvation shell. This observation is also corroborated by the analysis of trends in Br^−^⋯H–N RDFs presented above.

Ab initio molecular dynamics simulations, in contrast to MD simulations based on classical force fields, allow direct access to properties based on the electron density of the system evolving in time. Although their increased computational complexity makes AIMD simulations inferior to classical MD in terms of feasible simulation time or system size, this is compensated for by the increased knowledge of the system’s chemical reactivity and other properties related to its electron density [32]. In particular, calculating the centers of maximally localized Wannier functions (MLWF) provides access to a clear picture of the electron pair localization in the molecule, consistent with chemical intuition. This leads to directly available time-dependent dipole moments that can be used, among other things, for the calculation of infrared spectra.

**Figure 8:**
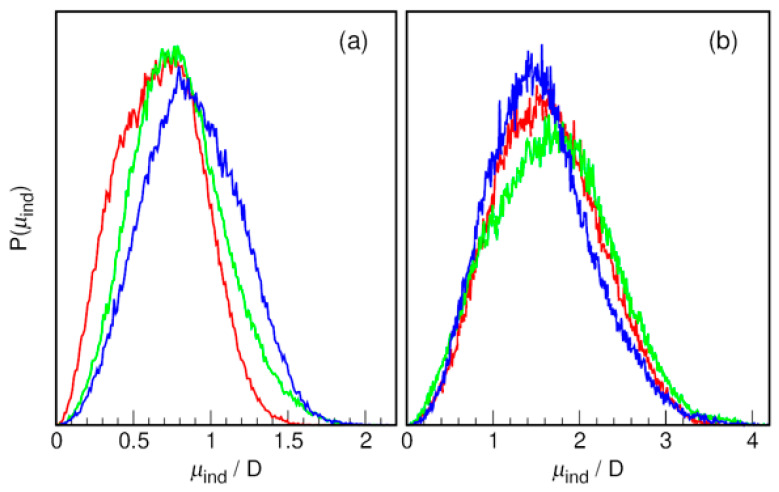
Induced dipole moments obtained from AIMD simulations for (**a**) Br^−^ anion and (**b**) tetrabutylammonium cation in DESs based on red—AP; green—MAE; and blue—BAE with an HBA–HBD molar ratio of 1:6.

In Figure 8, we present the probability distributions of the induced dipole moments of both ions in the studied TBAB-based DESs containing different alkanolamines. For the bromide ion, the most probable dipole moment value is about 0.75 D in AP- and MAE-based DESs, and this increases to about 0.8 D in the BAE-based DES. This value is comparable, for example, to that reported for the Cl^−^ anion in water, for which a dipole moment of 0.8 D has been reported [53]. Furthermore, the TBA^+^ cation is characterized by an induced dipole moment that is approximately twice as large and larger in MAE-based DES (ca. 1.6 D) than in the other two systems, which exhibit a maximum probability distribution of about 1.5 D. Clearly, both ions display a non-negligible induced dipole moment, resulting directly from the instantaneous polarization of their electron density by the solvent. As discussed below, this has a significant impact on the IR spectra of the studied DESs and shapes the ion–ion and ion–solvent interactions.

The above analysis aligns well with the determined Mulliken atomic charges of the ionic components, as shown in Table 1. Both ions possess charge lower than their formal charge, signifying that a non-negligible charge transfer is taking place in the DES. Notably, the charge redistribution is not balanced, and while the tetrabutylammonium cation retains about +0.8*e*, the anionic charge is only ca. −0.7*e*. The reason for this discrepancy is the non-negligible charge transfer to the solvent from the bromide anion, as evidenced by the average charge of −0.014*e* (for the AP molecule) and −0.02*e* (for secondary alkanolamines). Note that these are averaged values, and the actual charge redistribution is most probably concentrated on the solvent molecules hydrogen bonded to Br^−^. As a result, the bromide anion in the studied DES is much “softer” than expected based on its formal charge.

**Figure 9:**
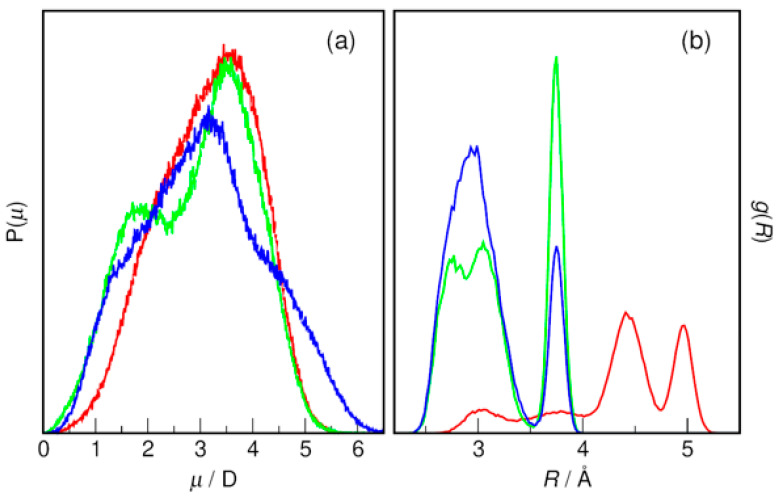
(**a**) Dipole moments of HBD and (**b**) intramolecular N⋯O radial distribution functions of HBD obtained from AIMD simulations in DESs based on red—AP; green—MAE; and blue—BAE with an HBA–HBD molar ratio of 1:6.

Alkanolamines are characterized by their own significant dipole moments in the studied DESs, as shown in Figure 9a. In contrast to the ions, the probability distributions clearly have a structure indicating the presence of several conformers with different mean dipole moments. This conformational flexibility of the HBD molecules is further confirmed by the intramolecular N⋯O RDFs shown in Figure 9b, which indicate the existence of elongated as well as convoluted molecules in the studied systems. AP, the only primary amine in the set, shows a clear tendency to take on stretched conformation with the two most probable forms characterized by an N⋯O distance of 4.4 Å and 5 Å, respectively. In the case of MAE and BAE, due to the smaller separation of N and O atoms in the molecule (by two methyl groups instead of three as in the case of AP), the longest possible N⋯O distance is shorter than 4 Å (RDF peak maximum at 3.75 Å). In contrast to AP molecules, they show much greater tendency to exist in the folded form, with the N⋯O distance in the range 2.5–3.5 Å. It should be noted that the formation of an intramolecular hydrogen bond is impossible here for steric reasons, but it is possible in the case of AP molecules, in which the conformational stress is much smaller.

**Figure 10:**
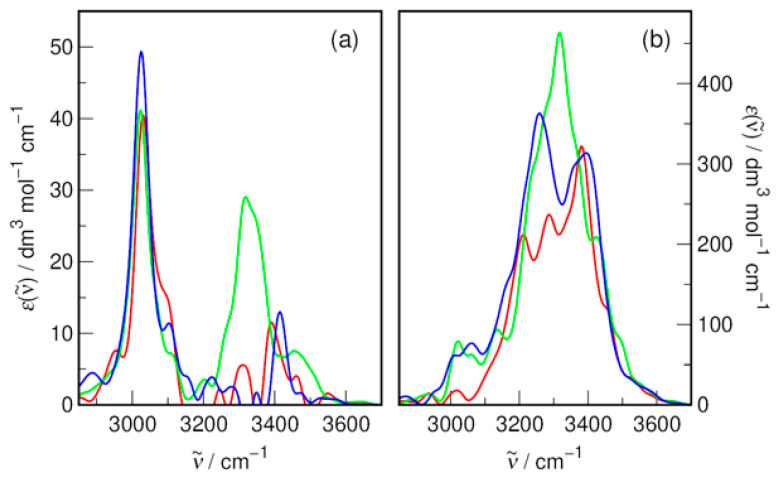
The infrared cross-spectra for (**a**) Br^−^–tetrabutylammonium and (**b**) Br^−^–HBD interactions obtained from AIMD simulations in DESs based on red—AP; green—MAE; and blue—BAE with an HBA–HBD molar ratio of 1:6.

The availability of time-dependent dipole moments allows us to calculate the infrared (IR) spectra of the studied DES. In particular, the cross-spectra between different molecule types deliver a lot of information about the intermolecular interactions [54]. It is worth noting that even monoatomic polarizable ions, which cannot show any meaningful vibrational spectrum of their own, contribute to the IR spectrum of the system via induced dipole moment effects, as first demonstrated for aqueous F^−^ [55]. The cross-IR spectra were obtained, as usual, by Fourier transforming the dipole moment cross-correlation function linking two different molecule types in the system and smoothing by passing through a Gaussian filter of 15 cm^−1^ width; see ref. [54] for details. The resulting spectra on a molar absorptivity scale (i.e., after dividing by the molar concentration of bromide in the system) are shown in Figure 10.

The Br^−^–tetrabutylammonium cross-IR spectra reveal a striking difference between the MAE-based DES and the other two alkanolamines shown in Figure 10a. While all spectra exhibit a similar intensity peak at ca. 3030 cm^−1^, resulting from interionic coupling with the C–H stretching vibrations of the TBA^+^ cation, the MAE-based system also reveals a high intensity peak at ~3320 cm^−1^, i.e., at the location of the O–H and N–H stretching vibrations of the solvent.

Only in this case is the solute–solvent vibrational coupling strong enough to influence the cation–anion coupling. On the other hand, the intensities of the first peaks in RDFs related to Br^−^⋯H–(O/N) interactions obtained from classical MD simulations change monotonically with (AP < MAE < BAE), and their maximum positions remain approximately constant, as shown in Figure 5 and Table S2. Therefore, it is clearly demonstrated that AIMD goes beyond the structural analysis itself and more deeply analyzes the mutual polarizability effects underlying the intermolecular interactions in the system.

The cross-Br^−^–HBD spectra shown in Figure 10b show considerable coupling of the bromide ion to the O–H and N–H stretching vibrations of alkanolamines, with negligible intensity in the expected C–H stretching vibration range. Thus, hydrogen bonds with solvent molecules decisively shape the induced dipole moment of bromide ions and make a significant contribution to the total IR spectrum of the systems. This is consistent with the results obtained earlier for other aqueous halides, for which the spectral intensity on the molar absorptivity scale for the ion IR spectrum was comparable to the solvent itself [53,55]. Again, the MAE-based system shows the highest intensity of the discussed peak, which is in agreement with the interionic cross-spectrum.

In terms of LAXS measurements, the organic molecules and ions in this study, 3-aminopropan-1-ol [56], 2-(methylamino)ethanol [57], 2-(*n*-butylamino)ethanol and tetrabutylammonium ion [58] have well-known structures. The C–C, C–O and C–N bond distances are ca. 1.515, 1.43, and 1.47 Å, respectively, and the C∙∙∙C, C∙∙∙O and C∙∙∙N distances are ca. 2.515, 2.45, and 2.46 Å, respectively. However, longer intramolecular distances in this type of molecule/ion cannot be observed by LAXS as such distances vary due to the free rotation around the single bonds in these molecules/ions.

The C–O and C–N distances in the solvent molecules and the tetrabutylammonium ion were treated as fixed parameters of 1.43 and 1.47 Å, respectively [58]. The C–C bond distance was refined to check validity of the assumed C–O and C–N distances. The C∙∙∙C, C∙∙∙O, and C∙∙∙N distances were refined to a single distance, as it is not possible to resolve them from each other. The only other short-range distances in these systems are the distances to the bromide ion, Br^−^∙∙∙O and Br^−^∙∙∙N. The hydrated bromide ion in aqueous solution binds on average six water molecules in basically octahedral fashion with a mean Br∙∙∙O distance of ca. 3.34 Å [59,60]. It can be expected that the Br^−^∙∙∙N distance is somewhat longer, as the atomic radius of nitrogen in amines (1.46 Å) is larger than oxygen in water and alcohols (1.35 Å) [61].

**Figure 11:**
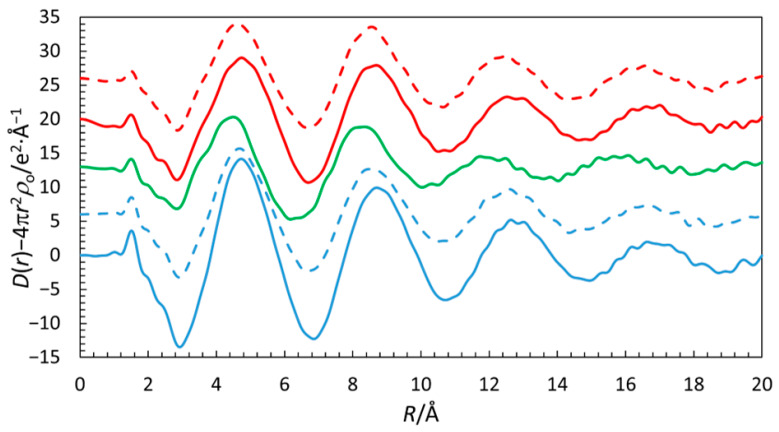
RDFs of the DES solutions from LAXS measurements, TBAB/AP 1:4 (red dashed line, offset +26), TBAB/AP 1:6 (red solid line, offset: +20), TBAB/MAE 1:6 (green line, offset: +13), TBAB/BAE 1:4 (blue dashed line, offset: +6), and TBAB/BAE 1:6 (blue solid line, no offset).

The RDFs of the five DESs studied by LAXS are very similar up to ca. 4 Å and all have remarkable intense peaks at ca. 4.7, 8.5, 12.5, and 16.6 Å, showing that the systems have very high internal structural order, as shown in Figure 11. The fits of the RDFs and intensity functions are given in Figures S2–S6 in the Supplementary Material. The refined structure parameters of the studied DES solutions are summarized in Table 2 and Table S6.

The mean Br^−^∙∙∙O distance in the DES, 3.31 Å, as shown in Table 2, is slightly shorter than in the hydrated bromide ion in aqueous solution, 3.34 Å, where it is six-coordinate in octahedral fashion [60,61]. The refined mean Br^−^∙∙∙N distance is within the expected range in comparison to the mean Br^−^∙∙∙O distance, vide supra. The observed Br^−^∙∙∙O and Br^−^∙∙∙N distances indicate a coordination number slightly below six, and this is further supported by the fact that no well-defined O∙∙∙O/N distances corresponding to an octahedral environment around the bromide ion are observed. The total number of Br^−^∙∙∙O and Br^−^∙∙∙N distances are set to 4.24 after simulation results, as shown in Table 2. It should be emphasized that the number of distances and temperature factor coefficients in LAXS studies are strongly correlated, and it is not possible to accurately determine the number of distances. The observed Br^−^∙∙∙O distance shows clearly that the total coordination number of the solvated bromide ion is less than six, and the applied total coordination number is taken from simulation studies. Upon increasing the total number of distances in the refinements, the temperature factor coefficient increases accordingly, but the distance remains.

**Figure 12:**
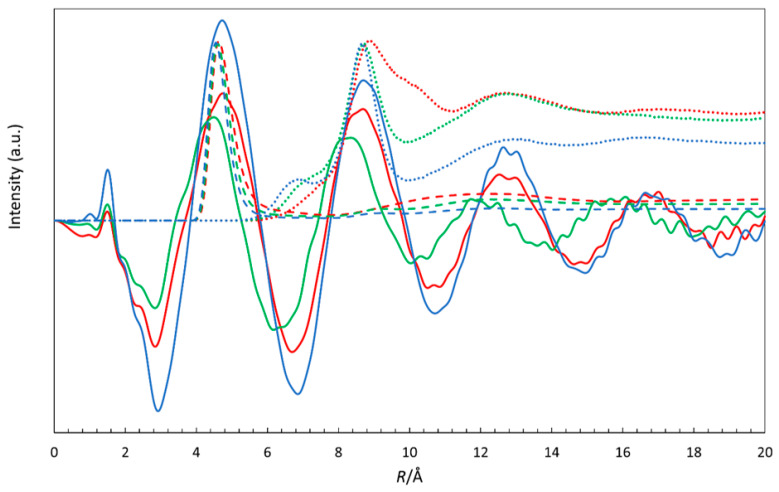
RDFs of the DES solutions in this study. TBAB/AP 1:6 (red), TBAB/MAE 1:6 (green), and TBAB/BAE 1:6 (blue) obtained from LAXS measurements (solid lines) together with arbitrarily scaled RDF curves obtained from molecular dynamics simulations for the N^+^∙∙∙Br^−^ distances (dashed lines) and Br^−^∙∙∙Br^−^ distances (dotted lines).

The most striking observation in the RDFs from the LAXS studies is the well-defined long-range structure with intense peaks at ca. 4.7, 8.5, 12.5, and 16.6 Å, as shown in Figure 11. These peaks are certainly in part related to Br^−^∙∙∙Br^−^ distances, but other well-defined distances such as N^+^∙∙∙Br^−^ also contribute. In order to confirm this hypothesis, we compared the LAXS curves with the RDF curves obtained from molecular dynamics for the 1:6 DES based on TBAB, as shown in Figure 12. The curves were rescaled in order to highlight the similarities between the methods and underline the oscillations visible above 4 Å. It is clear that while the first of these peaks (at ca. 4.7 Å) is attributable to the N^+^∙∙∙Br^−^ distances, the further ones are a result of long-range ordering of bromide anions in DESs. It is worth noting that the N^+^∙∙∙Br^−^ RDFs do not show significant structure beyond ca. 6 Å and that the Br^−^∙∙∙Br^−^ interactions decidedly contribute to the long-range ordering of the studied DESs.

## 3. Computational Methods

**Figure 13:**
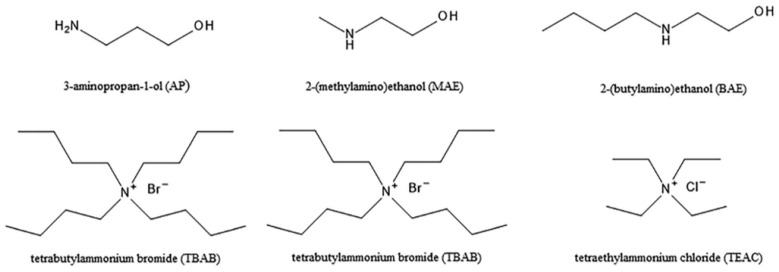
Chemical structures of the DES components used in this study.

*Molecular dynamics simulations.* MD simulations were carried out using the Gromacs package (version 2018.5) [62]. The general purpose all-atom flexible OPLS-AA force field was used, which is overall quite successful in reproducing the thermodynamic and structural properties of organic liquids alone and in mixtures with water [63,64]. It has also been successfully applied in studies of various DES systems in the recent past [30,65,66]. The cubic simulation boxes included a total of 945 molecules for DES systems and 1000 molecules for DES + water systems; see Table 3 for the systems’ composition. The systems were initially packed at experimental density using Gromacs solvation tools (gmx solvate and gmx insert-molecules). The chemical structures of all DES components are shown in Figure 13.

After initial energy minimization with a maximum force criterion of 100 kJ·mol^−1^·nm^−1^, each system was equilibrated for 5 ns in the isothermal–isobaric (*NPT*) ensemble, after which a further 50 ns *NPT* run was used for the production trajectory, during which data were collected for analysis. A time step of 0.5 fs was used throughout. The temperature was kept at 298 K by a canonical velocity rescaling thermostat [67], while pressure was stabilized at 1 bar using the Parrinello–Rahman barostat [68]. The time constants were set to 0.1 ps and 5 ps for the thermostat and the barostat, respectively. Analysis of results was performed using Gromacs package utilities (gmx rdf and gmx energy).

AIMD simulations were carried out using the cp2k computational suite (v. 6.0) [69,70,71]. Only the 1:6 TBAB/(AP, MAE, or BAE) systems were studied. The systems were composed of 5 TBAB ion pairs and 30 solvent molecules for AP or MAE, while 4 TBAB ion pairs and 24 solvent molecules were used for BAE. Each system was packed at experimental density using Gromacs solvation tools (gmx insert-molecules) and initially equilibrated for 5 ns in the canonical (*NVT*) ensemble in Gromacs using the simulation parameters as described above. The final configuration from this run was used as an input for further AIMD simulations.

The electronic structure was described using density functional theory (DFT), and the revPBE exchange-correlation functional [72,73] provided by the libXC library [74] was applied. Only valence electrons were represented explicitly in terms of Gaussian atomic orbitals and a plane waves (GPW) dual-basis set. The molecularly optimized short-range DZVP-MOLOPT-SR-GTH basis set of double zeta quality [75] was used for atomic orbitals, in combination with the auxiliary plane wave expansion of the electron density with cutoff set to 550 Ry. Core electrons were represented implicitly by norm-conserving GTH pseudopotentials [76] parameterized for the PBE functional, as using them gives essentially the same results as using reoptimized parameters [77]. The electron density and its derivative were smoothed on a spatial integration grid (using keywords XC_SMOOTH_RHO NN10 and XC_DERIV NN10_SMOOTH in cp2k), as it was previously found to provide an improved description of the potential energy surface for liquid systems [78]. The DFT energy was combined with the DFT-D3 empirical dispersion correction with Becke–Johnson damping [79,80] including only two-body terms and with the cutoff set to 15 Å.

Each system was initially equilibrated for ~30 ps in the *NVT* ensemble using a 0.5 fs time step. The temperature was set to 298 K by a canonical velocity rescaling thermostat [67] with a time constant set to 16.67 fs. After the equilibration period, the thermostat was removed and the production trajectory was further propagated in the microcanonical (*NVE*) ensemble for ca. 50 ps. Atomic coordinates and velocities were collected every 4 steps (i.e., every 2 fs), along with the centers of maximally localized Wannier functions (MLWF), which were later used to calculate dipole moments [81]. Mulliken atomic charges were calculated every 2 ps based on instantaneous wavefunction using the Multiwfn program (v. 3.8) [82,83].

## 4. Experimental Methods

*For large-angle X-ray scattering (LAXS) measurements*, mixtures of tetrabutylammonium bromide (TBAB) with 2-(methylamino)ethanol (MAE), 2-(*n*-butylamino)ethanol (BAE), and 3-aminopropan-1-ol (AP) with salt–solvent at molar ratios of 1:4 and 1:6 were studied; see Table 4 for sample data.

A large-angle *θ*–*θ* diffractometer was used to measure the scattering of MoKa radiation (*λ* = 0.7107 Å) on the free surfaces of the liquid samples. The solutions were contained in a Teflon cuvette with an airtight radiation shielding with beryllium windows. The scattered radiation was monochromatized in a focusing LiF crystal monochromator, and the intensity was measured at 450 discrete points in the range 1 < *θ* < 65° (the scattering angle was 2*θ*). A total of 100,000 counts were accumulated at each preset angle, and the whole angular range was scanned twice, which corresponds to a statistical error of about 0.3%. The divergence of the primary X-ray beam was limited by 1° or ¼° slits for different *θ* regions, with some parts of the data overlapping for scaling purposes. All of the data treatment was carried out with the KURVLR program [84]. All the details in the data treatment approach can be found elsewhere [85]. The experimental intensities were normalized to a stoichiometric unit of volume containing one bromine atom, using the scattering factors *f* for neutral atoms, including corrections for anomalous dispersion *Δf*′ and *Δf*″ [86] and values for Compton scattering [87,88]. Least squares refinements of the model parameters were carried out by means of the STEPLR program [89], where the expression *U* = Σ[*s*·*i*_exp_(*s*) − *s*·*i*_calc_(*s*)]^2^ was minimized. In order to obtain a better alignment of the intensity function before the refinements, a Fourier back-transformation procedure was used to correct the *i*_exp_(*s*) functions by removing spurious non-physical peaks below 1.2 Å in the experimental radial distribution function (RDF) [82]. Corrections due to the low absorption coefficients, *μ*, were applied [90].

DESs were prepared according to our previously published procedure, which was adopted from reported studies [2,46]. The 3-aminopropan-1-ol, 2-(*n*-butylamino)ethanol, 2-(methylamino)ethanol, and tetra-*n*-butyl-ammonium bromide were purchased from Sigma-Aldrich. TBAB was dried under reduced pressure at 323 K for 48 h before use, while other chemicals were used as supplied by the producer. Detailed information on the substances, including their purities, is provided in Table 5.

The solvents were prepared gravimetrically using appropriate amounts of each component, weighed with a Mettler Toledo balance (precision of 0.00001 g). The chemicals were thoroughly mixed at a temperature of 353.15 K for 1 h until a clear, homogeneous liquid without any precipitate was formed. The densities of the DES were measured at 293.15 K using a digital vibration-tube densimeter (Anton Paar DSA 5000, Graz, Austria) equipped with proportional temperature control, ensuring temperature stability within ±0.01 K. The instrument was calibrated using double-distilled, deionized, and degassed water and dry air at atmospheric pressure (0.1013 MPa).

## 5. Conclusions

The molecular structure and ion coordination of the tetraalkylammonium halide–alkanolamine DESs investigated in this work are a result of a complex interplay of hydrogen bonding, steric constraints, ion size, and significant polarization phenomena. Classical molecular dynamics simulations reveal that increasing the alkanolamine content weakens individual hydrogen bonds between the halide anion and the hydrogen bond donor, even as the total number of such contacts increases. This behavior is reflected in diminishing first-shell X^−^⋯H–N and X^−^⋯H–O peak intensities and rising coordination numbers, particularly in AP- and MAE-based systems. These structural features are consistent with experimentally observed viscosity trends, where lower salt concentrations promote weaker interacting liquid networks.

The identity of the hydrogen bond acceptor further modulates the DES microstructure. Chloride-based systems exhibit stronger and shorter hydrogen bond interactions than bromide-based analogues, whereas TBAB-containing DESs display more numerous but weaker N^+^⋯Br^−^ contacts. This balance leads to greater structural organization in TBAC-based liquids, while the higher molar mass of Br^−^ accounts for the elevated densities and refractive indices reported for TBAB systems. Variation in cation size additionally influences the liquid structure; DESs formed with a smaller TEA^+^ cation exhibit more defined solvation environments and higher coordination numbers than those containing TBA^+^, in agreement with their more ordered structural signatures and higher measured densities.

The type of hydrogen bond donor also has a pronounced effect on the microscopic organization of the studied DESs. AP, the only primary alkanolamine, yields two distinct X^−^⋯H–N coordination modes and forms the largest number of hydrogen bond contacts, creating a broad but relatively weak network. Secondary alkanolamines MAE and BAE form fewer but individually stronger interactions, with BAE showing the strongest X^−^⋯H contacts yet the lowest coordination numbers due to steric hindrance from its butyl substituent. These structural distinctions correlate directly with the measured densities and viscosities of the corresponding DESs.

Introducing water as an additional component of the system has a profound effect on the stability of TBAB-based DES. With increasing water content, the alkanolamine molecules are displaced from the bromide first solvation shell by water, and existing TBA + Br^−^ ion pairs are slowly replaced by hydrated individual ions. Bromide shows two well-defined hydration shells in all aqueous systems. Careful analysis of the number of contacts with bromide by water and alkanolamine allows one also to analyze preferential solvation in the DES. While the preferential solvation index of water is negative for AP-based systems (meaning that there is less water in the solvation shell than expected by random arrangement), the situation in DESs based on secondary alkanolamines is reversed, with water being more abundant than expected.

AIMD simulations complement this structural picture by demonstrating that polarization effects play a central role in determining intermolecular interactions. Both Br^−^ and TBA^+^ develop substantial induced dipole moments (approximately 0.75–0.8 D for the anion and 1.5–1.6 D for the cation), indicating that the ions exist as dynamically polarizable species rather than just static charge centers. Significant charge transfer from Br^−^ to neighboring alkanolamines makes the anion a softer base and modifies hydrogen bond strengths. The alkanolamines themselves exhibit broad dipole–moment distributions arising primarily from conformational flexibility. Polarization effects influence not only local solvation structure but also spectroscopic properties; IR cross-spectra reveal strong ion–ion vibrational coupling, particularly in MAE-based DES, underscoring the contribution of mutual polarization to the vibrational response and to the effective strength of intermolecular interactions.

Large-angle X-ray scattering measurements further confirm that all studied DESs possess a high degree of medium- and long-range organization, with pronounced peaks detectable up to 16.6 Å. Experimentally observed Br^−^⋯H–O and Br^−^⋯H–N distances agree with the MD predictions and are consistent with anion coordination numbers below six, reflecting a non-octahedral solvation environment. The persistence of long-range correlations likely arises from combined Br^−^⋯Br^−^ and N^+^⋯Br^−^ structuring, although their precise decomposition remains challenging. Comparison with results of MD simulations supports this interpretation and attributes the first pronounced LAXS peak to N^+^⋯Br^−^ interactions, while further long-range ordering is due to pronounced anion–anion interactions.

To recapitulate, this work demonstrates that the microscopic behavior of tetraalkylammonium halide–alkanolamine DESs is governed by the combined effects of hydrogen bonding, ion size and steric effects, and pronounced electronic polarization. The integration of classical MD, AIMD, and LAXS provides a detailed multi-faceted description of these systems. The insights derived from this combined approach offer a rational basis for fine-tuning DES composition, enabling predictive control over physicochemical properties relevant to both fundamental studies and practical applications.

## Figures and Tables

**Table 1:** Average Mulliken atomic charge per molecule (in units of elementary charge) obtained from AIMD simulations.

System	TBA^+^	Br^−^	Alkanolamines
TBAB/AP 1:6	0.789	−0.706	−0.014
TBAB/MAE 1:6	0.803	−0.685	−0.020
TBAB/BAE 1:6	0.808	−0.687	−0.020

**Table 2:** Mean bond distances to bromide, *d*/Å; number of distances, *n*; and temperature coefficients, *b*/Å^2^ in the LAXS studies of DES solvents at room temperature. The estimated standard deviations given within parentheses include only statistical errors. Numbers without parentheses were applied as fixed values.

Interaction	TBAB/AP 1:4	TBAB/AP 1:6	TBAB/MAE 1:6	TBAB/BAE 1:4	TBAB/BAE 1:6
*d* (Br∙∙∙O)	3.312 (7)	3.294 (7)	3.309 (6)	3.312 (6)	3.315 (8)
*b* (Br∙∙∙O)	0.0119 (10)	0.0125 (10)	0.0120 (8)	0.0104 (9)	0.0132 (10)
*n* (Br∙∙∙O)	2.47	2.47	2.47	2.47	2.47
*d* (Br∙∙∙N)	3.470 (13)	3.470 (13)	3.461 (14)	3.471 (12)	3.478 (12)
*b* (Br∙∙∙N)	0.0129 (18)	0.0134 (8)	0.0136 (18)	0.0119 (17)	0.0143 (23)
*n* (Br∙∙∙N)	1.77	1.77	1.77	1.77	1.77

**Table 3:** Number of molecules in the cubic simulation box for each system.

HBA/HBD Molar Ratio	TBA^+^/TEA^+^	Br^−^/Cl^−^	AP/MAE/BAE	H_2_O
1:4	189	189	756	-
1:6	135	135	810	-
1:8	105	105	840	-
1:10	85	85	860	-
Aqueous DES solutions composed of TBAB and AP/MAE/BAE with a molar ratio of 1:6				
x_H2O_ = 0.1	129	129	771	100
x_H2O_ = 0.2	114	114	686	200
x_H2O_ = 0.3	100	100	600	300
x_H2O_ = 0.4	86	86	514	400
x_H2O_ = 0.5	71	71	429	500
x_H2O_ = 0.6	57	57	343	600
x_H2O_ = 0.7	43	43	257	700
x_H2O_ = 0.8	29	29	171	800
x_H2O_ = 0.9	14	14	86	900

**Table 4:** Compositions (in mol·dm^−3^), densities at 293.15 K (*ρ*), linear absorption coefficients (*μ*), solvent to salt ratio, x, and water content in mass fraction of the tetrabutylammonium bromide solutions used in the LAXS experiments.

System	[TBA^+^]	[Br^−^]	[Solvent]	*ρ*/g·cm^−3^	*μ*/cm^−1^	Water Content ^a^
TBAB/AP 1:4	1.638	1.638	6.552	1.02017	11.076	0.00066
TBAB/AP 1:6	1.309	1.309	7.854	1.01190	9.008	0.00053
TBAB/MAE 1:6	1.276	1.276	7.653	0.98606	8.788	0.00154
TBAB/BAE 1:4	1.203	1.203	4.813	0.95193	8.256	0.00078
TBAB/BAE 1:6	0.914	0.914	5.484	0.93731	6.425	0.00059

^a^ Water content of DESs in mass fraction determined by Karl Fisher titration with the standard uncertainty ±0.0001.

**Table 5:** Chemicals used and their CAS numbers and purities as stated by the supplier. All chemicals were supplied by Sigma-Aldrich.

IUPAC Chemical Name	Formula	CAS No.	Purity/Mass Fraction
3-Aminopropan-1-ol	H_2_N(CH_2_)_2_CH_2_OH	111-75-1	≥0.98
2-(Methylamino)ethanol	CH_3_NHCH_2_CH_2_OH	109-83-1	≥0.98
2-(*n*-Butylamino)ethanol	*n*-C_4_H_9_NHCH_2_CH_2_OH	111-75-1	≥0.98
Tetra-*n*-butylammonium bromide	(*n*-C_4_H_9_)_4_NBr	1643-19-2	≥0.99

## Data Availability

The data supporting the results are available from the corresponding author on request.

## Supplementary Materials

The following supporting information can be downloaded at: https://www.mdpi.com/article/10.3390/molecules31081364/s1. Table S1. The comparison of experimental densities of the studied DES systems at 298 K with the values obtained from MD simulations. Table S2. The location of first maxima and first minima of radial distribution functions, as well as coordination number values, for the studied deep eutectic solvents based on TBAB, obtained from MD simulations. The reference site is Br^−^. Table S3. The location of first maxima and first minima of radial distribution functions, as well as coordination number values for the studied deep eutectic solvents based on TEAC, obtained from MD simulations. The reference site is Cl^−^. Table S4. The location of first maxima and first minima of radial distribution functions, as well as coordination number values for the studied deep eutectic solvents based on TBAC, obtained from MD simulations. The reference site is Cl^−^. Figure S1. A snapshot of a single bromide ion with its coordination environment from the trajectory of the TBAB/MAE 1:6 system. The bromide ion is shown as the large pink sphere, the TBA cations in ball and stick representation, and the solvent molecules in stick representation. Hydrogen bonds shown as black dashed lines. The solvent forms three hydrogen bonds with Br– and several intermolecular MAE–MAE hydrogen bonds are also visible. At the same time, the central anion is coordinated by two TBA cations with alkyl chains displaced in order to lower the Br–⋯N+ distance. Table S5. The location of first maxima and first minima of radial distribution functions, as well as coordination number values, for the studied aqueous mixtures of deep eutectic solvents based on TBAB, obtained from MD simulations. The reference site is Br^−^. Table S6. Additional mean bond distances, d/Å, number of distances, n, and temperature coefficients, b/Å2, in the LAXS studies of DES solvents at room temperature. The estimated standard deviations given within parentheses include only statistical errors. Numbers without parentheses were applied as fixed values. Figure S2. (Top) LAXS radial distribution functions for Bu4NBr:3-aminopropan-1-ol (1:4), AP14. Upper part: Experimental RDF, D(r) − 4πr2ρo (black line), sum of model contributions (red line); difference (blue line). Lower part Reduced LAXS intensity functions s.i(s) (black line); model s.icalc(s) (red line). Figure S3. (Top) LAXS radial distribution functions for Bu4NBr:3-aminopropan-1-ol (1:6), AP16. Upper part: Experimental RDF, D(r) − 4πr2ρo (black line), sum of model contributions (red line); difference (blue line). Lower part Reduced LAXS intensity functions s.i(s) (black line); model s.icalc(s) (red line). Figure S4. (Top) LAXS radial distribution functions for Bu4NBr:2-methylamino-ethanol (1:6), MAE16. Upper part: Experimental RDF, D(r) − 4πr2ρo (black line), sum of model contributions (red line); difference (blue line). Lower part Reduced LAXS intensity functions s.i(s) (black line); model s.icalc(s) (red line). Figure S5. (Top) LAXS radial distribution functions for Bu4NBr:2-(butylamino)ethanol (1:4), BAE14. Upper part: Experimental RDF, D(r) − 4πr2ρo (black line), sum of model contributions (red line); difference (blue line). Lower part Reduced LAXS intensity functions s.i(s) (black line); model s.icalc(s) (red line). Figure S6. (Top) LAXS radial distribution functions for Bu4NBr:2-(butylamino)ethanol (1:6), BAE16. Upper part: Experimental RDF, D(r) − 4πr2ρo (black line), sum of model contributions (red line); difference (blue line). Lower part Reduced LAXS intensity functions s.i(s) (black line); model s.icalc(s) (red line).

## References

[1] Zhang L., Wang M. (2017). Optimization of deep eutectic solvent-based ultrasound-assisted extraction of polysaccharides from Dioscorea opposita Thunb. Int. J. Biol. Macromol..

[2] Abbott A.P., Capper G., Gray S. (2006). Design of Improved Deep Eutectic Solvents Using Hole Theory. ChemPhysChem.

[3] Martins M.A.R., Pinho S.P., Coutinho J.A.P. (2019). Insights into the Nature of Eutectic and Deep Eutectic Mixtures. J. Solut. Chem..

[4] Hansen B.B., Spittle S., Chen B., Poe D., Zhang Y., Klein J.M., Horton A., Adhikari L., Zelovich T., Doherty B.W. (2021). Deep Eutectic Solvents: A Review of Fundamentals and Applications. Chem. Rev..

[5] Jenkin G.R.T., Al-Bassam A.Z.M., Harris R.C., Abbott A.P., Smith D.J., Holwell D.A., Chapman R.J., Stanley C.J. (2016). The application of deep eutectic solvent ionic liquids for environmentally-friendly dissolution and recovery of precious metals. Miner. Eng..

[6] Malaquias J.C., Steichen M., Thomassey M., Dale P.J. (2013). Electrodeposition of Cu–In alloys from a choline chloride based deep eutectic solvent for photovoltaic applications. Electrochim. Acta.

[7] Schaeffer N., Martins M.A.R., Neves C.M.S.S., Pinho S.P., Coutinho J.A.P. (2018). Sustainable hydrophobic terpene-based eutectic solvents for the extraction and separation of metals. Chem. Commun..

[8] Nowosielski B., Warmińska D., Cichowska-Kopczyńska I. (2023). CO_2_ Separation Using Supported Deep Eutectic Liquid Membranes Based on 1,2-propanediol. ACS Sustain. Chem. Eng..

[9] Xie Y., Dong H., Zhang S., Lu X., Ji X. (2016). Solubilities of CO_2_, CH_4_, H_2_, CO and N_2_ in choline chloride/urea. Green Energy Environ..

[10] Zhao H., Baker G.A., Holmes S. (2011). Protease activation in glycerol-based deep eutectic solvents. J. Mol. Catal. B Enzym..

[11] Tolmachev D., Lukasheva N., Ramazanov R., Nazarychev V., Borzdun N., Volgin I., Andreeva M., Glova A., Melnikova S., Dobrovskiy A. (2022). Computer Simulations of Deep Eutectic Solvents: Challenges, Solutions, and Perspectives. Int. J. Mol. Sci..

[12] Velez C., Acevedo O. (2022). Simulation of deep eutectic solvents: Progress to promises. WIREs Comput. Mol. Sci..

[13] Kaur S., Kumari M., Kashyap H.K. (2020). Microstructure of Deep Eutectic Solvents: Current Understanding and Challenges. J. Phys. Chem. B.

[14] Kovács A., Neyts E.C., Cornet I., Wijnants M., Billen P. (2020). Modeling the Physicochemical Properties of Natural Deep Eutectic Solvents. ChemSusChem.

[15] Sun H., Li Y., Wu X., Li G. (2013). Theoretical study on the structures and properties of mixtures of urea and choline chloride. J. Mol. Model..

[16] Perkins S.L., Painter P., Colina C.M. (2014). Experimental and computational studies of choline chloride-based deep eutectic solvents. J. Chem. Eng. Data.

[17] Naik P.K., Paul S., Banerjee T. (2019). Physiochemical Properties and Molecular Dynamics Simulations of Phosphonium and Ammonium Based Deep Eutectic Solvents. J. Solut. Chem..

[18] Migliorati V., Sessa F., D’Angelo P. (2019). Deep eutectic solvents: A structural point of view on the role of the cation. Chem. Phys. Lett. X.

[19] Ferreira E.S.C., Voroshylova I.V., Figueiredo N.M., Cordeiro M.N.D.S. (2021). Molecular dynamic study of alcohol-based deep eutectic solvents. J. Chem. Phys..

[20] Khorasani Z.T., Bihoudipour Y., Monhemi H., Vahidi S.H., Eftekhari M. (2025). Molecular dynamics simulation of polyethylene glycol-based deep eutectic solvents: Microstructure, dynamics and potentials for curcumin delivery. J. Mol. Liq..

[21] Rozas S., Alomari N., Atilhan M., Aparicio S. (2021). Theoretical insights into the cineole-based deep eutectic solvents. J. Chem. Phys..

[22] Kussainova D., Shah D. (2019). Structure of monoethanolamine based type III DESs: Insights from molecular dynamics simulations. Fluid Phase Equilib..

[23] Barani Pour S., Jahanbin Sardroodi J., Rastkar Ebrahimzadeh A. (2021). The study of structure and interactions of glucose-based natural deep eutectic solvents by molecular dynamics simulation. J. Mol. Liq..

[24] He N., Chen Q., Fan J., Song F., Dong N. (2023). In-depth theoretical study on the structures of betaine-1,2-propanediol based deep eutectic solvents. J. Mol. Liq..

[25] Lane J.N., Klimkowski V.J., Hopkins T.A. (2025). Molecular dynamics investigation of deep eutectic solvent structure and properties based on hydrogen bond acceptor variation. J. Mol. Liq..

[26] Shah D., Mjalli F.S. (2014). Effect of water on the thermo-physical properties of Reline: An experimental and molecular simulation based approach. Phys. Chem. Chem. Phys..

[27] Zhekenov T., Toksanbayev N., Kazakbayeva Z., Shah D., Mjalli F.S. (2017). Formation of type III Deep Eutectic Solvents and effect of water on their intermolecular interactions. Fluid Phase Equilib..

[28] Aravena P., Cea-Klapp E., Gajardo-Parra N.F., Held C., Garrido J.M., Canales R.I. (2023). Effect of water and hydrogen bond acceptor on the density and viscosity of glycol-based eutectic solvents. J. Mol. Liq..

[29] Monteiro H., Santos A., Sarmento C., Farinha A., Witkamp G.J., Paiva A., Ferreira A.S.D., Duarte A.R.C., Galamba N. (2026). Water-induced modifications in the physicochemical and structural properties of betaine and choline chloride—Ethylene glycol deep eutectic solvents. J. Mol. Liq..

[30] Monteiro H., Paiva A., Duarte A.R.C., Galamba N. (2022). Structure and Dynamic Properties of a Glycerol–Betaine Deep Eutectic Solvent: When Does a DES Become an Aqueous Solution?. ACS Sustain. Chem. Eng..

[31] Garmroodi M.V., Sardroodi J.J., Bonab P.J., Verpoort F., Matin A.A. (2025). The effect of increasing water on the physicochemical features of thymol: DL-menthol based natural deep eutectic solvent (DES). Mater. Today Chem..

[32] Kirchner B., di Dio P.J., Hutter J. (2012). Real-World Predictions from Ab Initio Molecular Dynamics Simulations. Top. Curr. Chem..

[33] Zahn S., Kirchner B., Mollenhauer D. (2016). Charge Spreading in Deep Eutectic Solvents. ChemPhysChem.

[34] Korotkevich A., Firaha D.S., Padua A.A.H., Kirchner B. (2017). Ab initio molecular dynamics simulations of SO_2_ solvation in choline chloride/glycerol deep eutectic solvent. Fluid Phase Equilib..

[35] Zahn S. (2017). Deep eutectic solvents: Similia similibus solvuntur?. Phys. Chem. Chem. Phys..

[36] Fetisov E.O., Harwood D.B., Kuo I.F.W., Warrag S.E.E., Kroon M.C., Peters C.J., Siepmann J.I. (2018). First-Principles Molecular Dynamics Study of a Deep Eutectic Solvent: Choline Chloride/Urea and Its Mixture with Water. J. Phys. Chem. B.

[37] Alizadeh V., Malberg F., Pádua A.A.H., Kirchner B. (2020). Are There Magic Compositions in Deep Eutectic Solvents? Effects of Composition and Water Content in Choline Chloride/Ethylene Glycol from Ab Initio Molecular Dynamics. J. Phys. Chem. B.

[38] Alizadeh V., Esser L., Kirchner B. (2021). How is CO_2_ absorbed into a deep eutectic solvent?. J. Chem. Phys..

[39] Malik A., Dhattarwal H.S., Kashyap H.K. (2021). Distinct Solvation Structures of CO_2_ and SO_2_ in Reline and Ethaline Deep Eutectic Solvents Revealed by AIMD Simulations. J. Phys. Chem. B.

[40] Spittle S., Poe D., Doherty B., Kolodziej C., Heroux L., Haque M.A., Squire H., Cosby T., Zhang Y., Fraenza C. (2022). Evolution of microscopic heterogeneity and dynamics in choline chloride-based deep eutectic solvents. Nat. Commun..

[41] Malik A., Kashyap H.K. (2022). Solvent Organization around Methane Dissolved in Archetypal Reline and Ethaline Deep Eutectic Solvents as Revealed by AIMD Investigation. J. Phys. Chem. B.

[42] Busato M., Di Lisio V., Del Giudice A., Tomai P., Migliorati V., Galantini L., Gentili A., Martinelli A., D’Angelo P. (2021). Transition from molecular- to nano-scale segregation in a deep eutectic solvent—Water mixture. J. Mol. Liq..

[43] Di Muzio S., Russina O., Mastrippolito D., Benassi P., Rossi L., Paolone A., Ramondo F. (2022). Mixtures of choline chloride and tetrabutylammonium bromide with imidazole as examples of deep eutectic solvents: Their structure by theoretical and experimental investigation. J. Mol. Liq..

[44] Chai K., Zhou Y., Lu X., Yamaguchi T., Ohara K., Liu H., Zhu F. (2023). Structure of choline chloride-carboxylic acid deep eutectic solvents by wide-angle X-ray scattering and DFT calculations. Phys. Chem. Chem. Phys..

[45] Jahanbakhsh-Bonab P., Heidaryan E., Motallebzadeh M., Sardroodi J.J. (2026). Assessment of the nanostructural and physicochemical properties of amine-based deep eutectic solvents for extraction processes. Results Eng..

[46] Nowosielski B., Jamrógiewicz M., Łuczak J., Tercjak A., Warmińska D. (2023). Effect of temperature and composition on physical properties of deep eutectic solvents based on 2-(methylamino)ethanol—measurement and prediction. J. Mol. Liq..

[47] Nowosielski B., Jamrógiewicz M., Łuczak J., Śmiechowski M., Warmińska D. (2020). Experimental and predicted physicochemical properties of monopropanolamine-based deep eutectic solvents. J. Mol. Liq..

[48] Nowosielski B., Jamrógiewicz M., Cichowska-Kopczyńska I., Warmińska D. (2024). Comprehensive evaluation of physical properties and carbon dioxide capacities of new 2-(butylamino)ethanol-based deep eutectic solvents. Pure Appl. Chem..

[49] Yusof R., Jumbri K., Abdul Rahman M.B. (2021). An insight into the effects of ratios and temperatures on a tetrabutylammonium bromide and ethylene glycol deep eutectic solvent. J. Mol. Liq..

[50] Migliorati V., D’Angelo P. (2021). Deep eutectic solvents: A structural point of view on the role of the anion. Chem. Phys. Lett..

[51] Salehi H.S., Celebi A.T., Vlugt T.J.H., Moultos O.A. (2021). Thermodynamic, transport, and structural properties of hydrophobic deep eutectic solvents composed of tetraalkylammonium chloride and decanoic acid. J. Chem. Phys..

[52] Panda D.K., Bhargava B.L. (2022). Effect of hydration on intermolecular interactions in tetrabutylammonium chloride based deep eutectic solvents. J. Mol. Liq..

[53] Śmiechowski M. (2018). Unusual Influence of Fluorinated Anions on the Stretching Vibrations of Liquid Water. J. Phys. Chem. B.

[54] Śmiechowski M. (2021). Molecular level interpretation of excess infrared spectroscopy. J. Mol. Liq..

[55] Smiechowski M., Forbert H., Marx D. (2013). Spatial decomposition and assignment of infrared spectra of simple ions in water from mid-infrared to THz frequencies: Li+(aq) and F-(aq). J. Chem. Phys..

[56] Gajda R., Katrusiak A. (2008). Pressure-freezing with conformational conversion of 3-aminopropan-1-ol molecules. Acta Crystallogr. Sect. B Struct. Sci..

[57] Nishikiori S., Takahashi-Ebisudani Y., Iwamoto T. (1990). Novel series of clathrate compounds of the three-dimensional metal complex hosts (*N*-methyl-1,3-diaminopropane)cadmium(II) tetracyanonickelate(II), (*N*, *N*-Dimethyl-1,3-diaminopropane)cadmium(II) Tetracyanonickelate(II), and (2-Hydroxyethylmethylamine) cadmi. J. Incl. Phenom. Mol. Recognit. Chem..

[58] Groom C.R., Bruno I.J., Lightfoot M.P., Ward S.C. (2016). The Cambridge structural database. Acta Crystallogr. Sect. B Struct. Sci. Cryst. Eng. Mater..

[59] Jalilehvand F., Spångberg D., Lindqvist-Reis P., Hermansson K., Persson I., Sandström M. (2001). Hydration of the calcium ion. An EXAFS, large-angle X-ray scattering, and molecular dynamics simulation study. J. Am. Chem. Soc..

[60] Dangelo P., Migliorati V., Guidoni L. (2010). Hydration properties of the bromide aqua ion: The interplay of first principle and classical molecular dynamics, and X-ray absorption spectroscopy. Inorg. Chem..

[61] Shannon R.D. (1976). Revised effective ionic radii and systematic studies of interatomic distances in halides and chalcogenides. Acta Crystallogr. Sect. A.

[62] Abraham M.J., Murtola T., Schulz R., Páll S., Smith J.C., Hess B., Lindah E. (2015). Gromacs: High performance molecular simulations through multi-level parallelism from laptops to supercomputers. SoftwareX.

[63] Jorgensen W.L., Maxwell D.S., Tirado-Rives J. (1996). Development and testing of the OPLS all-atom force field on conformational energetics and properties of organic liquids. J. Am. Chem. Soc..

[64] Kaminski G., Duffy E.M., Matsui T., Jorgensen W.L. (1994). Free energies of hydration and pure liquid properties of hydrocarbons from the OPLS all-atom model. J. Phys. Chem..

[65] Panda D.K., Bhargava B.L. (2021). Intermolecular interactions in tetrabutylammonium chloride based deep eutectic solvents: Classical molecular dynamics studies. J. Mol. Liq..

[66] Kivelä H., Salomäki M., Vainikka P., Mäkilä E., Poletti F., Ruggeri S., Terzi F., Lukkari J. (2022). Effect of Water on a Hydrophobic Deep Eutectic Solvent. J. Phys. Chem. B.

[67] Bussi G., Donadio D., Parrinello M. (2007). Canonical sampling through velocity rescaling. J. Chem. Phys..

[68] Parrinello M., Rahman A. (1981). Polymorphic transitions in single crystals: A new molecular dynamics method. J. Appl. Phys..

[69] The cp2k Developers Group *cp2k*, version 6.0. https://cp2k.org.

[70] Hutter J., Iannuzzi M., Schiffmann F., VandeVondele J. (2014). cp2k: Atomistic simulations of condensed matter systems. WIREs Comput. Mol. Sci..

[71] Kühne T.D., Iannuzzi M., Del Ben M., Rybkin V.V., Seewald P., Stein F., Laino T., Khaliullin R.Z., Schütt O., Schiffmann F. (2020). CP_2_K: An electronic structure and molecular dynamics software package—Quickstep: Efficient and accurate electronic structure calculations. J. Chem. Phys..

[72] Zhang Y., Yang W. (1998). Comment on “Generalized Gradient Approximation Made Simple”. Phys. Rev. Lett..

[73] Perdew J.P., Burke K., Ernzerhof M. (1996). Generalized Gradient Approximation Made Simple. Phys. Rev. Lett..

[74] Marques M.A.L., Oliveira M.J.T., Burnus T. (2012). Libxc: A library of exchange and correlation functionals for density functional theory. Comput. Phys. Commun..

[75] VandeVondele J., Hutter J. (2007). Gaussian basis sets for accurate calculations on molecular systems in gas and condensed phases. J. Chem. Phys..

[76] Goedecker S., Teter M., Hutter J. (1996). Separable dual-space Gaussian pseudopotentials. Phys. Rev. B.

[77] Ruiz Pestana L., Mardirossian N., Head-Gordon M., Head-Gordon T. (2017). Ab initio molecular dynamics simulations of liquid water using high quality meta-GGA functionals. Chem. Sci..

[78] Jonchiere R., Seitsonen A.P., Ferlat G., Saitta A.M., Vuilleumier R. (2011). Van der Waals effects in ab initio water at ambient and supercritical conditions. J. Chem. Phys..

[79] Grimme S., Antony J., Ehrlich S., Krieg H. (2010). A consistent and accurate ab initio parametrization of density functional dispersion correction (DFT-D) for the 94 elements H-Pu. J. Chem. Phys..

[80] Grimme S., Ehrlich S., Goerigk L. (2011). Effect of the damping function in dispersion corrected density functional theory. J. Comput. Chem..

[81] Marzari N., Vanderbilt D. (1997). Maximally localized generalized Wannier functions for composite energy bands. Phys. Rev. B.

[82] Lu T., Chen F. (2012). Multiwfn: A multifunctional wavefunction analyzer. J. Comput. Chem..

[83] Lu T. (2024). A comprehensive electron wavefunction analysis toolbox for chemists, Multiwfn. J. Chem. Phys..

[84] Johansson G. (1973). Computer Programs for the Analysis of Data on X-ray Diffraction by Liquids. Chem. Scr..

[85] Stålhandske C.M.V., Persson I., Sandström M., Kamienska-Piotrowicz E. (1997). Structure of the Solvated Zinc(II), Cadmium(II), and Mercury(II) Ions in *N*, *N*-Dimethylthioformamide Solution. Inorg. Chem..

[86] Ibers J.A., Hamilton W.C. (1983). International Tables for X-Ray Crystallography.

[87] Cromer D. (1969). Compton scattering factors for aspherical free atoms. J. Chem. Phys..

[88] Cromer D.T., Mann J.B. (1967). Compton Scattering Factors for Spherically Symmetric Free Atoms. J. Chem. Phys..

[89] Molund M., Persson I. (1985). STEPLR-a program for refinements of data on X-ray-scattering by liquids. Chem. Scr..

[90] Levy H.A., Danford M.D., Narten A.H. (1966). Data Collection and Evaluation with an X-Ray Diffractometer Designed for the Study of Liquid Structure.

