# Supplementary material for: Molecular Insight into the Structural Properties of Deep Eutectic Solvents Based on Alkanolamines—A Theoretical and Experimental Study
Source: Molecules. 2026 Apr 21;31(8):1364. doi: 10.3390/molecules31081364 (PMC13118984; doi:10.3390/molecules31081364)
Supplement: Supplementary file 1 [file molecules-31-01364-s001.zip › molecules-4187608-supplementary.pdf]

Supplementary Materials

Molecular Insight into Structural Properties of the Deep Eutectic Solvents Based on Alkanolamines – a theoretical and experimental study

Maciej Śmiechowski[a*], Bartosz Nowosielski[b], Ingmar Persson[c], Iwona Cichowska-Kopczyńska[d], Dorota Warmińska[a]

[a] Department of Physical Chemistry, Faculty of Chemistry, Gdańsk University of Technology, ul. Narutowicza 11/12, 80-233 Gdańsk, POLAND

[b] Maritime Advanced Research Centre, Szczecińska 65, 80-392 Gdańsk, POLAND

[c] Department of Molecular Sciences, Swedish University of Agricultural Sciences, P.O. Box 7015, SE-750 07 Uppsala, SWEDEN

[d] Department of Process Engineering and Chemical Technology, Faculty of Chemistry, Gdańsk University of Technology, ul. Narutowicza 11/12, 80-233 Gdańsk, POLAND

[*]Corresponding author: Tel.:+485834711283; fax:+48583472694,
e-mail address: maciej.smiechowski@pg.edu.pl

Table S1. The comparison of experimental densities of the studied DES systems at 298 K with the values obtained from MD simulations.

	Experimental [kg·m^{-3}]			MD [kg·m^{-3}]			Relative error [%]		
Salt/Solvent	AP[a]	MAE[b]	BAE[c]	AP	MAE	BAE	AP	MAE	BAE
TBAB 1:4	1018.5			1001.6			1.7		
TBAB 1:6	1011.9	982.4	934.0	1000.9	969.5	915.7	1.1	1.3	2.0
TBAB 1:8	1007.1	974.4	925.2	1000.1	965.7	908.7	0.7	0.9	1.8
TBAB 1:10		969.6	918.8		960.9	903.5		0.9	1.7
TEAC 1:4	995.1			987.1			0.8		
TEAC 1:6	992.4	955.5	908.0	983.7	949.2	892.5	0.9	0.7	1.7
TEAC 1:8	990.8	952.4	904.1	976.9	946.9	889.5	1.4	0.6	1.6
TEAC 1:10		950.2	901.3		945.4	886.8		0.5	1.6
TBAC 1:4	958.0			935.3			2.4		
TBAC 1:6	963.4	936.2	897.7	947.9	926.3	879.7	1.6	1.1	2.0
TBAC 1:8	966.9	936.8	896.0	956.8	922.0	879.6	1.0	1.6	1.8
TBAC 1:10		936.4	894.6		919.1	879.2		1.8	1.7

[a] B. Nowosielski, M. Jamrógiewicz, J. Łuczak, M. Śmiechowski, D. Warmińska, Experimental and predicted physicochemical properties of monopropanolamine-based deep eutectic solvents, J. Mol. Liq. 309 (2020) 113110.
[b] B. Nowosielski, M. Jamrógiewicz, J. Łuczak, A. Tercjak, D. Warmińska, Effect of temperature and composition on physical properties of deep eutectic solvents based on 2-(methylamino)ethanol – measurement and prediction, J. Mol. Liq. 371 (2023) 121069, 1-14.
[c] B. Nowosielski, M. Jamrógiewicz, I. Cichowska-Kopczyńska, D. Warmińska, Comprehensive evaluation of physical properties and carbon dioxide capacities of new 2-(butylamino)ethanol-based deep eutectic solvents, Pure Appl. Chem. 96 (2024) 1733–1749.

Table S2. The location of first maxima and first minima of radial distribution functions, as well as coordination number values, for the studied deep eutectic solvents based on TBAB, obtained from MD simulations. The reference site is Br^-.

site[a]	r_{max}[b]	r_{min}[c]	N_c[d]	site[a]	r_{max}[b]	r_{min}[c]	N_c[d]	site[a]	r_{max}[b]	r_{min}[c]	N_c[d]
	TBAB/AP 1:4				TBAB/MAE 1:6				TBAB/BAE 1:6		
HN	0.250	0.462	3.566	HN	0.244	0.354	0.836	HN	0.244	0.352	1.039
HO	0.232	0.344	2.145	HO	0.232	0.340	2.208	HO	0.232	0.338	1.757
N^+	0.460	0.776	2.118	N^+	0.456	0.76	1.891	N^+	0.448	0.758	1.689
	TBAB/AP 1:6				TBAB/MAE 1:8				TBAB/BAE 1:8		
HN	0.250	0.454	3.857	HN	0.244	0.348	0.878	HN	0.244	0.352	1.108
HO	0.232	0.344	2.467	HO	0.232	0.340	2.434	HO	0.232	0.336	1.896
N^+	0.458	0.768	1.812	N^+	0.454	0.742	1.669	N^+	0.452	0.756	1.562
	TBAB/AP 1:8				TBAB/MAE 1:10				TBAB/BAE 1:10		
HN	0.252	0.452	3.997	HN	0.244	0.352	0.904	HN	0.246	0.352	1.163
HO	0.232	0.338	2.641	HO	0.232	0.342	2.520	HO	0.232	0.336	2.060
N^+	0.460	0.788	1.685	N^+	0.456	0.776	1.626	N^+	0.450	0.776	1.556

[a] Observed atom. [b] Position of maximum [nm]. [c] Position of minimum [nm]. [d] Integration of first peak.

Table S3. The location of first maxima and first minima of radial distribution functions, as well as coordination number values for the studied deep eutectic solvents based on TEAC, obtained from MD simulations. The reference site is Cl^-.

site[a]	r_{max}[b]	r_{min}[c]	N_c[d]	site[a]	r_{max}[b]	r_{min}[c]	N_c[d]	site[a]	r_{max}[b]	r_{min}[c]	N_c[d]
	TEAC/AP 1:4				TEAC/MAE 1:6				TEAC/BAE 1:6		
HN	0.246	0.446	3.583	HN	0.238	0.348	0.856	HN	0.238	0.350	1.103
HO	0.226	0.334	2.299	HO	0.226	0.332	2.475	HO	0.226	0.334	2.035
N^+	0.458	0.746	2.698	N^+	0.456	0.740	2.411	N^+	0.450	0.698	2.084
	TEAC/AP 1:6				TEAC/MAE 1:8				TEAC/BAE 1:8		
HN	0.246	0.446	3.978	HN	0.238	0.346	0.891	HN	0.238	0.346	1.111
HO	0.226	0.332	2.677	HO	0.226	0.332	2.599	HO	0.226	0.330	2.195
N^+	0.462	0.766	2.299	N^+	0.456	0.742	2.209	N^+	0.452	0.730	1.981
	TEAC/AP 1:8				TEAC/MAE 1:10				TEAC/BAE 1:10		
HN	0.246	0.444	4.112	HN	0.238	0.348	0.934	HN	0.238	0.350	1.139
HO	0.226	0.338	2.866	HO	0.226	0.332	2.790	HO	0.226	0.334	2.277
N^+	0.458	0.768	2.042	N^+	0.454	0.746	1.964	N^+	0.450	0.636	1.778

[a] Observed atom. [b] Position of maximum [nm]. [c] Position of minimum [nm]. [d] Integration of first peak.

Table S4. The location of first maxima and first minima of radial distribution functions, as well as coordination number values for the studied deep eutectic solvents based on TBAC, obtained from MD simulations. The reference site is Cl^-.

site[a]	r_{max}[b]	r_{min}[c]	N_c[d]	site[a]	r_{max}[b]	r_{min}[c]	N_c[d]	site[a]	r_{max}[b]	r_{min}[c]	N_c[d]
	TBAC/AP 1:4				TBAC/MAE 1:6				TBAC/BAE 1:6		
HN	0.244	0.450	3.432	HN	0.238	0.342	0.884	HN	0.238	0.352	1.025
HO	0.226	0.340	2.265	HO	0.226	0.332	2.369	HO	0.226	0.332	1.953
N^+	0.454	0.772	2.095	N^+	0.450	0.752	1.795	N^+	0.446	0.748	1.714
	TBAC/AP 1:6				TBAC/MAE 1:8				TBAC/BAE 1:8		
HN	0.244	0.446	3.710	HN	0.238	0.340	0.890	HN	0.240	0.350	1.054
HO	0.226	0.336	2.626	HO	0.226	0.332	2.616	HO	0.226	0.332	2.232
N^+	0.454	0.782	1.823	N^+	0.452	0.756	1.622	N^+	0.448	0.754	1.561
	TBAC/AP 1:8				TBAC/MAE 1:10				TBAC/BAE 1:10		
HN	0.246	0.442	3.840	HN	0.238	0.346	0.891	HN	0.240	0.346	1.138
HO	0.226	0.338	2.820	HO	0.226	0.336	2.758	HO	0.226	0.332	2.281
N^+	0.456	0.768	1.602	N^+	0.450	0.768	1.429	N^+	0.444	0.762	1.370

[a] Observed atom. [b] Position of maximum [nm]. [c] Position of minimum [nm]. [d] Integration of first peak.

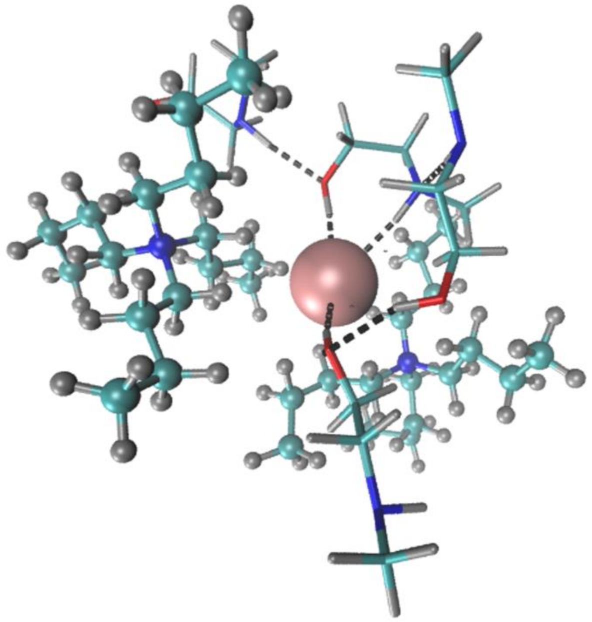

Figure S1. A snapshot of a single bromide ion with its coordination environment from the trajectory of the TBAB/MAE 1:6 system. The bromide ion is shown as the large pink sphere, the TBA cations in ball and stick representation, and the solvent molecules in stick representation. Hydrogen bonds shown as black dashed lines. The solvent forms three hydrogen bonds with Br^- and several intermolecular MAE–MAE hydrogen bonds are also visible. At the same time, the central anion is coordinated by two TBA cations with alkyl chains displaced in order to lower the $Br^- \cdots N^+$ distance.

Table S5. The location of first maxima and first minima of radial distribution functions, as well as coordination number values, for the studied aqueous mixtures of deep eutectic solvents based on TBAB, obtained from MD simulations. The reference site is Br^{-}.

site[a]	r_{max}^{b}	r_{min}^{c}	N_c^{d}	site[a]	r_{max}^{b}	r_{min}^{c}	N_c^{d}	site[a]	r_{max}^{b}	r_{min}^{c}	N_c^{d}
TBAB/AP 1:6, x_{DES} = 0.1				TBAB/MAE 1:6, x_{DES} = 0.1				TBAB/BAE 1:6, x_{DES} = 0.1			
HN	0.256	0.456	0.982	HN	0.254	0.314	0.078	HN	0.252	0.328	0.024
HO_{AA}^{e}	0.236	0.314	0.188	HO_{AA}^{e}	0.242	0.312	0.135	HO_{AA}^{e}	0.232	0.306	0.015
HO_{W}^{f}	0.228	0.302	6.028	HO_{W}^{f}	0.228	0.300	6.084	HO_{W}^{f}	0.226	0.300	6.296
N^{+}	0.486	0.598	0.151	N^{+}	0.482	0.564	0.112	N^{+}	0.486	0.610	0.163
TBAB/AP 1:6, x_{DES} = 0.2				TBAB/MAE 1:6, x_{DES} = 0.2				TBAB/BAE 1:6, x_{DES} = 0.2			
HN	0.252	0.478	2.145	HN	0.250	0.324	0.153	HN	0.252	0.330	0.065
HO_{AA}^{e}	0.236	0.318	0.421	HO_{AA}^{e}	0.236	0.310	0.300	HO_{AA}^{e}	0.234	0.308	0.131
HO_{W}^{f}	0.226	0.300	5.281	HO_{W}^{f}	0.228	0.300	5.550	HO_{W}^{f}	0.226	0.298	5.838
N^{+}	0.484	0.604	0.399	N^{+}	0.478	0.624	0.388	N^{+}	0.484	0.608	0.386
TBAB/AP 1:6, x_{DES} = 0.3				TBAB/MAE 1:6, x_{DES} = 0.3				TBAB/BAE 1:6, x_{DES} = 0.3			
HN	0.250	0.478	2.866	HN	0.250	0.326	0.267	HN	0.250	0.330	0.121
HO_{AA}^{e}	0.234	0.322	0.672	HO_{AA}^{e}	0.234	0.318	0.397	HO_{AA}^{e}	0.232	0.314	0.256
HO_{W}^{f}	0.228	0.298	4.428	HO_{W}^{f}	0.228	0.298	4.888	HO_{W}^{f}	0.228	0.298	5.256
N^{+}	0.484	0.626	0.737	N^{+}	0.482	0.636	0.728	N^{+}	0.472	0.616	0.656
TBAB/AP 1:6, x_{DES} = 0.4				TBAB/MAE 1:6, x_{DES} = 0.4				TBAB/BAE 1:6, x_{DES} = 0.4			
HN	0.254	0.476	3.249	HN	0.248	0.332	0.350	HN	0.248	0.334	0.222
HO_{AA}^{e}	0.232	0.326	0.969	HO_{AA}^{e}	0.232	0.318	0.697	HO_{AA}^{e}	0.234	0.322	0.413
HO_{W}^{f}	0.228	0.298	3.567	HO_{W}^{f}	0.228	0.298	4.094	HO_{W}^{f}	0.228	0.298	4.455
N^{+}	0.482	0.640	1.044	N^{+}	0.472	0.630	0.931	N^{+}	0.464	0.632	1.071
TBAB/AP 1:6, x_{DES} = 0.5				TBAB/MAE 1:6, x_{DES} = 0.5				TBAB/BAE 1:6, x_{DES} = 0.5			
HN	0.254	0.468	3.402	HN	0.246	0.336	0.463	HN	0.246	0.338	0.326
HO_{AA}^{e}	0.232	0.328	1.227	HO_{AA}^{e}	0.232	0.330	1.049	HO_{AA}^{e}	0.234	0.326	0.580
HO_{W}^{f}	0.226	0.298	2.811	HO_{W}^{f}	0.228	0.296	3.139	HO_{W}^{f}	0.226	0.298	3.695
N^{+}	0.478	0.658	1.269	N^{+}	0.464	0.664	1.278	N^{+}	0.462	0.662	1.382
TBAB/AP 1:6, x_{DES} = 0.6				TBAB/MAE 1:6, x_{DES} = 0.6				TBAB/BAE 1:6, x_{DES} = 0.6			
HN	0.252	0.468	3.632	HN	0.246	0.338	0.598	HN	0.246	0.338	0.459
HO_{AA}^{e}	0.232	0.330	1.553	HO_{AA}^{e}	0.234	0.332	1.277	HO_{AA}^{e}	0.234	0.328	0.782
HO_{W}^{f}	0.226	0.298	2.076	HO_{W}^{f}	0.228	0.296	2.379	HO_{W}^{f}	0.226	0.296	2.870
N^{+}	0.478	0.682	1.443	N^{+}	0.458	0.662	1.403	N^{+}	0.460	0.66	1.530
TBAB/AP 1:6, x_{DES} = 0.7				TBAB/MAE 1:6, x_{DES} = 0.7				TBAB/BAE 1:6, x_{DES} = 0.7			
HN	0.252	0.464	3.625	HN	0.246	0.342	0.725	HN	0.248	0.346	0.649
HO_{AA}^{e}	0.232	0.336	1.832	HO_{AA}^{e}	0.232	0.332	1.556	HO_{AA}^{e}	0.232	0.328	1.058
HO_{W}^{f}	0.226	0.298	1.423	HO_{W}^{f}	0.228	0.296	1.701	HO_{W}^{f}	0.226	0.296	2.055
N^{+}	0.474	0.682	1.565	N^{+}	0.458	0.660	1.459	N^{+}	0.456	0.674	1.621
TBAB/AP 1:6, x_{DES} = 0.8				TBAB/MAE 1:6, x_{DES} = 0.8				TBAB/BAE 1:6, x_{DES} = 0.8			
HN	0.252	0.460	3.681	HN	0.244	0.348	0.792	HN	0.246	0.346	0.802
HO_{AA}^{e}	0.232	0.340	2.035	HO_{AA}^{e}	0.232	0.338	1.843	HO_{AA}^{e}	0.232	0.332	1.330
HO_{W}^{f}	0.226	0.298	0.902	HO_{W}^{f}	0.226	0.296	1.058	HO_{W}^{f}	0.226	0.296	1.239
N^{+}	0.474	0.664	1.611	N^{+}	0.462	0.664	1.567	N^{+}	0.456	0.67	1.632
TBAB/AP 1:6, x_{DES} = 0.9				TBAB/MAE 1:6, x_{DES} = 0.9				TBAB/BAE 1:6, x_{DES} = 0.9			
HN	0.252	0.458	3.761	HN	0.246	0.350	0.939	HN	0.244	0.354	0.929
HO_{AA}^{e}	0.232	0.338	2.296	HO_{AA}^{e}	0.234	0.338	2.023	HO_{AA}^{e}	0.232	0.334	1.491

HO_W[f]	0.226	0.296	0.402	HO_W[f]	0.228	0.296	0.494	HO_W[f]	0.226	0.296	0.587
N^+	0.474	0.648	1.586	N^+	0.456	0.648	1.570	N^+	0.454	0.654	1.698

[a] Observed atom. [b] Position of maximum [nm]. [c] Position of minimum [nm]. [d] Integration of first peak. [e] Hydroxyl hydrogen of alkanolamine. [f] Water hydrogen.

Table S6. Additional mean bond distances, d/Å, number of distances, n, and temperature coefficients, b/Å^2, in the LAXS studies of DES solvents at room temperature. The estimated standard deviations given within parentheses include only statistical errors. Numbers without parentheses were applied as fixed values.

Interaction	TBAB/AP 1:4	TBAB/AP 1:6	TBAB/MAE 1:6	TBAB/BAE 1:4	TBAB/BAE 1:6
d(C–C)	1.511(2)	1.513(2)	1.509(4)	1.514(2)	1.513(2)
b(C–C)	0.0012(2)	0.0014(2)	0.0022(4)	0.0018(2)	0.0017(2)
n(C–C)	20	24	18	24	30
d(N–C)	1.470	1.470	1.470	1.470	1.470
b(N–C)	0.0015	0.0015	0.0015	0.0015	0.0015
n(N–C)	8	10	16	12	16
d(O–C)	1.430	1.430	1.430	1.430	1.430
b(O–C)	0.0015	0.0015	0.0015	0.0015	0.0015
n(O–C)	4	6	6	4	6
d(O–H)	0.960	0.960	0.960	0.960	0.960
b(O–H)	0.0010	0.0010	0.0010	0.0010	0.0010
n(O–H)	4	6	6	6	6
d(N–H)	0.960	0.960	0.960	0.960	0.960
b(N–H)	0.0010	0.0010	0.0010	0.0010	0.0010
n(N–H)	8	12	6	4	6
d(C–(C)–C/O/N)	2.509(5)	2.514(4)	2.518(8)	2.514(4)	2.517(2)
b(C–(C)–C/O/N)	0.0072(7)	0.0046(4)	0.0066(9)	0.0062(5)	0.0055(5)
m(C–(C)–C/O/N)	24	30	30	36	48

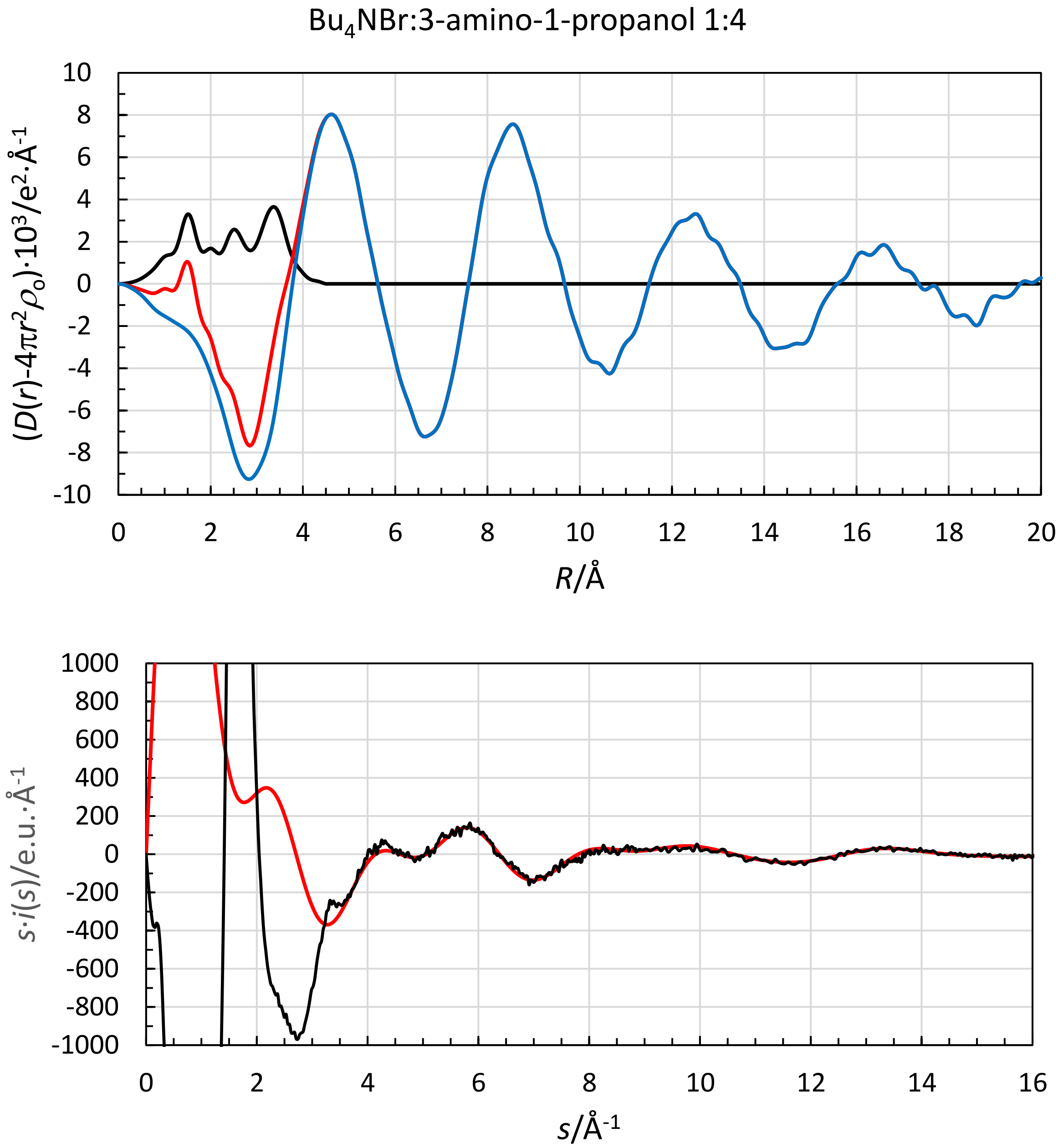

Figure S2. (Top) LAXS radial distribution functions for Bu_4NBr:3-aminopropan-1-ol (1:4), AP14. Upper part: Experimental RDF, $D(r) - 4\pi r^2\rho_o$ (black line), sum of model contributions (red line); difference (blue line). Lower part Reduced LAXS intensity functions $s{\cdot}i(s)$ (black line); model $s{\cdot}i_{calc}(s)$ (red line).

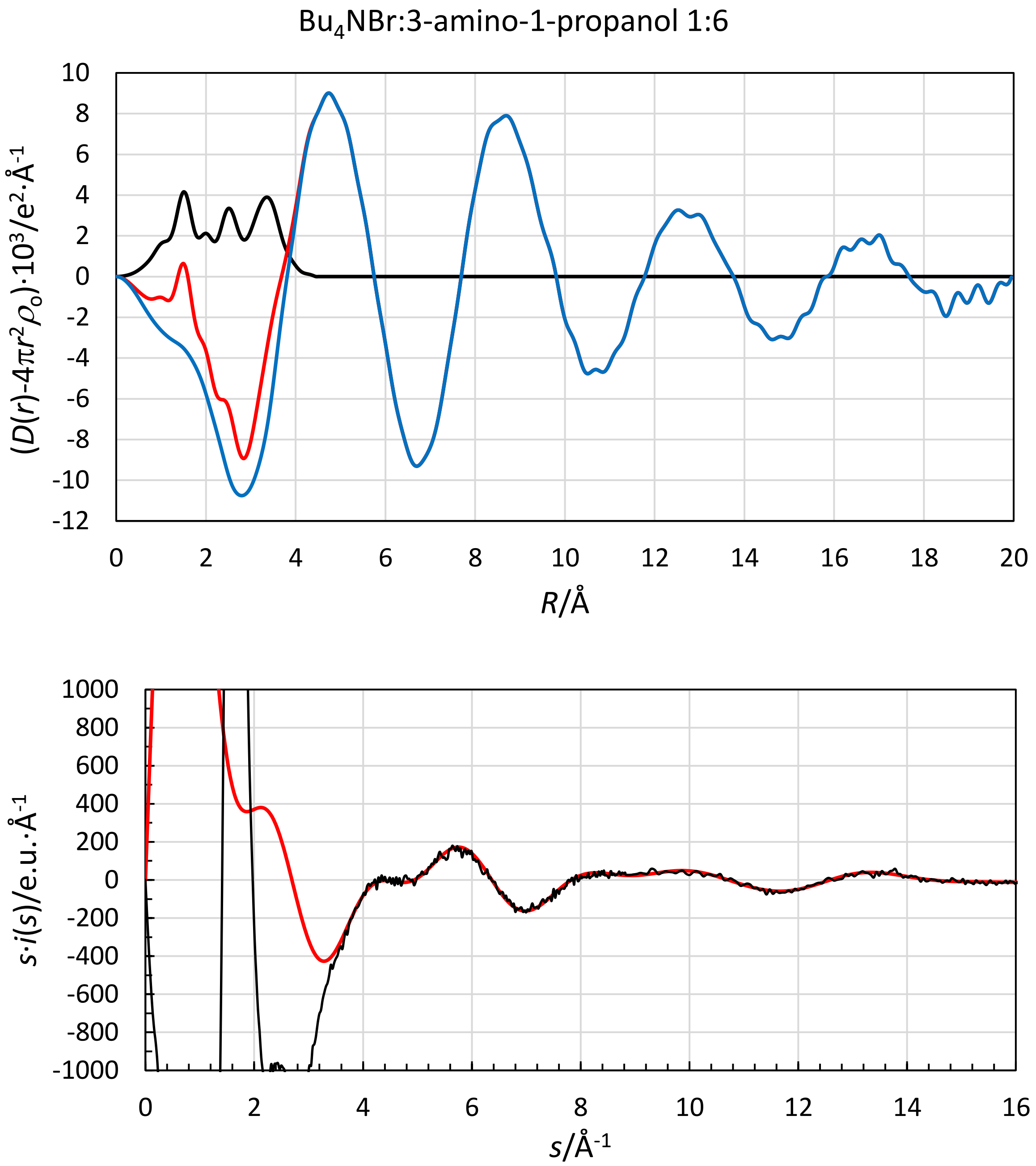

Figure S3. (Top) LAXS radial distribution functions for Bu_4NBr:3-aminopropan-1-ol (1:6), AP16. Upper part: Experimental RDF, $D(r) - 4\pi r^2\rho_o$ (black line), sum of model contributions (red line); difference (blue line). Lower part Reduced LAXS intensity functions $s{\cdot}i(s)$ (black line); model $s{\cdot}i_{calc}(s)$ (red line).

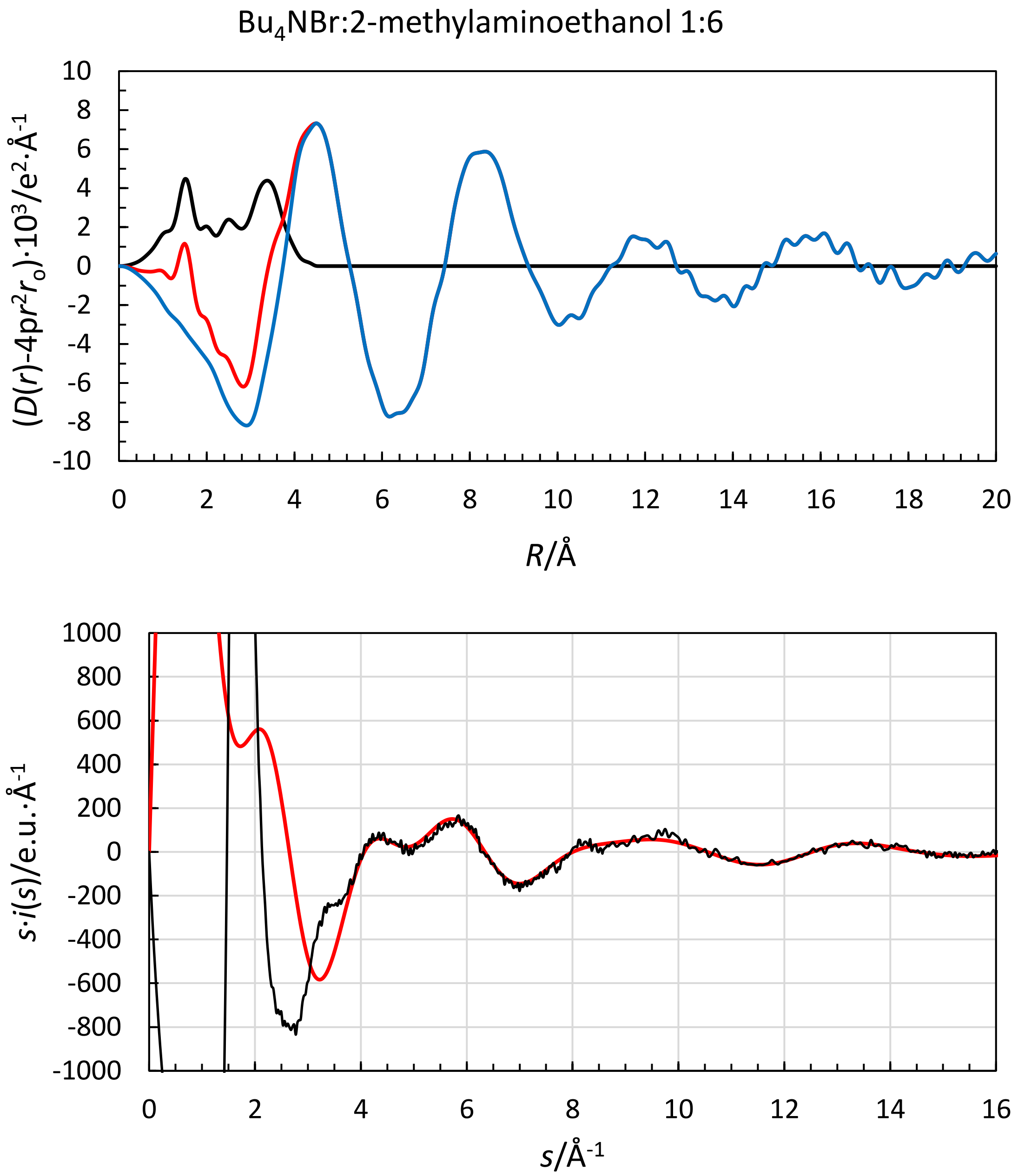

Figure S4. (Top) LAXS radial distribution functions for Bu_4NBr:2-methylamino-ethanol (1:6), MAE16. Upper part: Experimental RDF, $D(r) - 4\pi r^2\rho_o$ (black line), sum of model contributions (red line); difference (blue line). Lower part Reduced LAXS intensity functions $s\cdot i(s)$ (black line); model $s\cdot i_{calc}(s)$ (red line).

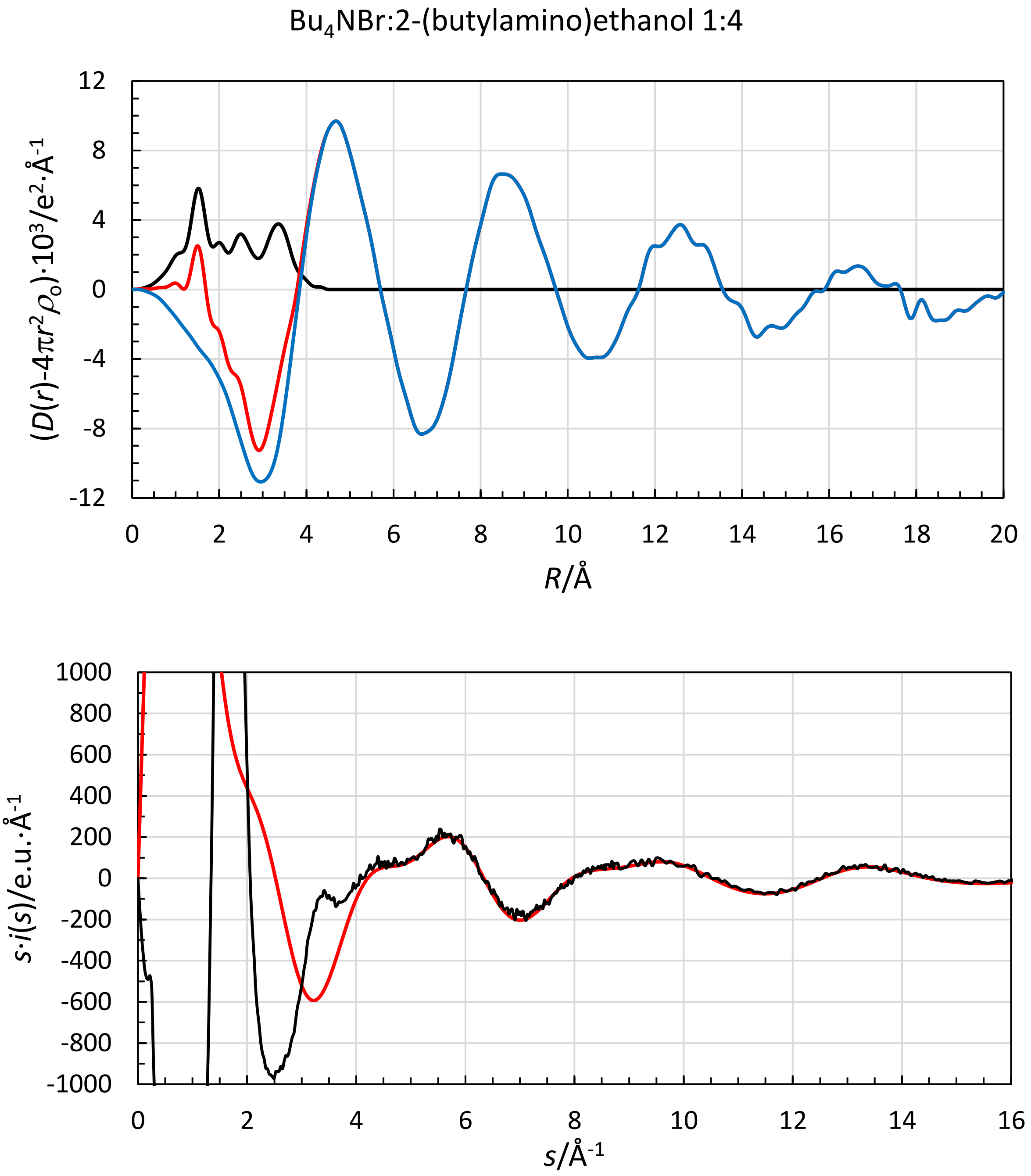

Figure S5. (Top) LAXS radial distribution functions for Bu_4NBr:2-(butylamino)ethanol (1:4), BAE14. Upper part: Experimental RDF, $D(r) - 4\pi r^2\rho_o$ (black line), sum of model contributions (red line); difference (blue line). Lower part Reduced LAXS intensity functions $s{\cdot}i(s)$ (black line); model $s{\cdot}i_{calc}(s)$ (red line).

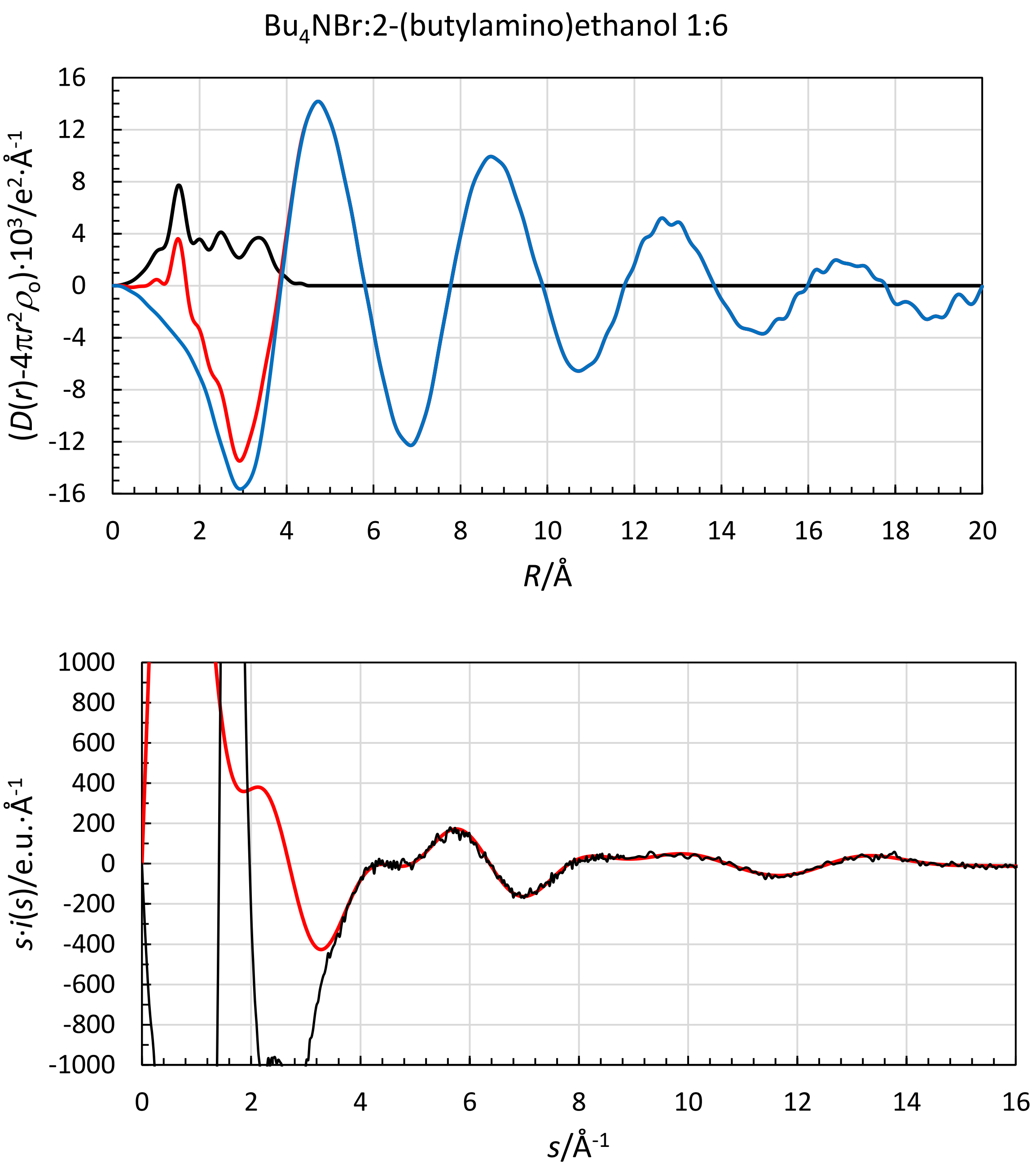

Figure S6. (Top) LAXS radial distribution functions for Bu_4NBr:2-(butylamino)ethanol (1:6), BAE16. Upper part: Experimental RDF, $D(r) - 4\pi r^2\rho_o$ (black line), sum of model contributions (red line); difference (blue line). Lower part Reduced LAXS intensity functions $s{\cdot}i(s)$ (black line); model $s{\cdot}i_{calc}(s)$ (red line).
